# Homeopathy for the treatment of insomnia: a systematic review and meta-analysis

**DOI:** 10.3389/frsle.2026.1922807

**Published:** 2026-09-28

**Authors:** Abhijit Dutta, Dewan Md. Sumsuzzman, Daniel Wrzałko, Robbert van Haselen, Petra Weiermayer, Katharina Gaertner, J. A. D. S Lakshani, Sabine Klein, Stephan Baumgartner, Martin Loef

**Affiliations:** 1 International Cooperation Section, Ministry of Ayush, New Delhi, India; 2 Agent-Based Modeling Laboratory, York University, Toronto, ON, Canada; 3 Institute of Complementary and Integrative Medicine, University of Bern, Bern, Switzerland; 4 Society for Clinical Research, Berlin, Germany; 5 International Institute for Integrated Medicine, London, United Kingdom; 6 Institute of Integrative Medicine, University of Witten/Herdecke, Witten, Germany; 7 Aarepraxis Bern, Bern, Switzerland; 8 Department of Information Technology, Faculty of Information Technology, University of Moratuwa, Moratuwa, Sri Lanka

**Keywords:** homeopathy, insomnia, meta-analysis, sleep disorders, systematic review

## Abstract

**Background:**

Insomnia is a prevalent disorder associated with substantial impairment. Homeopathy has been proposed as a complementary treatment for insomnia, but its clinical effects remain uncertain. This systematic review assessed the efficacy, effectiveness, and safety of homeopathic treatments for insomnia.

**Methods:**

Prospective comparative studies evaluating any homeopathic preparation for insomnia were included. Searches in MEDLINE, EMBASE, seven additional databases, and three trial registries were conducted through August 2025. Risk of bias, intervention complexity, model validity, and pragmatism were assessed using respectively RoB 2, ROBINS-I, iCAT, MVHT, and RITES. Data were synthesized using random-effects meta-analyses, and certainty of evidence was evaluated using GRADE.

**Results:**

Eight randomized controlled trials (RCTs; *n* = 364 participants) and four non-randomized studies on interventions (NRSIs; n = 517) met the inclusion criteria. In adults, sleep quality (MD = −2.6 points; 95% CI −5.5 to 0.26; low certainty) and insomnia severity (MD = −3.2; 95% CI −5.68 to −0.72, moderate certainty) were reported in one RCT each. For total sleep time, the pooled MD of three RCTs was 0.65 hours (95% CI −0.9 to 2.2; low certainty). In children, one open-label RCT suggested a difference in insomnia severity, but certainty of evidence was very low. Adverse events were rarely reported, resulting in low certainty evidence.

**Conclusions:**

The current evidence is mainly limited by imprecision and risk of bias. The available evidence does not allow firm conclusions regarding the effects of homeopathy for insomnia. High-quality, replicated trials with systematic adverse-event monitoring are needed.

## 1 Introduction

Insomnia is a prevalent and disabling sleep disorder defined as persistent difficulty with sleep initiation, maintenance, or early-morning awakening despite adequate sleep opportunity, causing significant daytime distress and impairment (American Academy of Sleep Medicine, 2014; Seow et al., 2018; American Psychiatric Association, 2022). Recent estimates suggest that insomnia affects 16.2% of adults globally, with 7.9% experiencing severe insomnia, and the highest burden observed among older adults, women, and individuals with comorbid medical or psychiatric conditions (Morin and Jarrin, 2022; Benjafield et al., 2025). Beyond individual suffering, chronic insomnia is increasingly recognized both as a clinical and a public health issue, contributing to impaired cognitive and physical functioning, increased risks for cardiovascular and metabolic diseases, reduced quality of life, and substantial societal costs including work absenteeism, productivity loss, and elevated healthcare utilization (Daley et al., 2009; Benjafield et al., 2025; Olatunde and Patton, 2025).

Clinical management of insomnia remains challenging despite advances in understanding its pathophysiology and an expanding array of interventions. Pharmacologic therapies, notably benzodiazepine receptor agonists, are commonly prescribed but raise concerns about tolerance, dependence, residual sedation, and cognitive impairment with long-term use (Morin and Jarrin, 2022). Cognitive behavioral therapy for insomnia (CBT-I) is recommended as the first-line treatment, but implementation is hampered by restricted access, patient adherence barriers, and gradual response times (Natsky et al., 2020). These limitations have spurred interest in alternative or complementary therapies that may offer safer, accessible, and effective options for those who are refractory to, or prefer, nonpharmacologic strategies.

Homeopathy, based on the principle of “treat likes by likes” and individualized use of highly diluted substances (Schmidt, 2021), is an alternative approach for insomnia treatment. Earlier animal studies reported changes in sleep-related outcomes following homeopathic preparations, including EEG-derived sleep measures in rats and alcohol-induced sleeping time in mice (Ruiz-Vega et al., 2002; Sukul et al., 1999). Although these models do not directly represent human insomnia or establish clinical efficacy, they provide complementary experimental context for research on sleep-related outcomes in humans. The clinical evidence base remains inconsistent and subject to methodological criticism, such as small sample sizes, population heterogeneity, variable homeopathic preparations, and outcome measure inconsistency (Cooper and Relton, 2010).

Current interest in integrating traditional, complementary and integrative medicine modalities into mainstream care, underscores the need for a rigorous, up-to-date evidence synthesis employing comprehensive search strategies and robust bias assessment tools. Earlier systematic reviews concluded that evidence for homeopathy in insomnia was unconvincing, but these reviews were conducted more than a decade ago and did not include quantitative synthesis or more recent trials and gray literature (Cooper and Relton, 2010; Ernst, 2011). Accordingly, this systematic review and outcome-specific meta-analysis updates and extends earlier syntheses to evaluate the efficacy, effectiveness and safety of homeopathic interventions for insomnia, adopting a complex systems perspective that incorporates patient involvement, model validity, and efficacy–effectiveness considerations (Van Haselen et al., 2026).

## 2 Methods

This systematic review was conducted in accordance with the methodological guidance provided in the *Cochrane Handbook for Systematic Reviews of Interventions* (Higgins et al., 2024). Reporting followed the Preferred Reporting Items for Systematic Reviews and Meta-Analyses (PRISMA) 2020 statement (Page et al., 2021), the PRISMA-S extension for literature searches (Rethlefsen et al., 2021), and the GRIPP2 guideline for patient involvement (Staniszewska et al., 2017). The review protocol was prospectively registered in the PROSPERO database (registration number: CRD420251015627) in combination with a template protocol (Loef et al., 2026). The present review adopts the template protocol in full (Loef et al., 2025), with insomnia-specific adaptations (see Supplementary File 1).

### 2.1 Literature search and study selection

On February, 21st, 2025 (last update by rerunning all searches: 13.8.2025), we conducted a systematic literature search according to chapter 4 of the Cochrane Handbook of Systematic Reviews (Lefebvre et al., 2024) and the PRESS peer review guideline (Mcgowan et al., 2016); one experienced reviewer (ML) and an information specialist (SK) were involved in the process. We searched databases (the HOMIS database (Gaertner et al., 2023), MEDLINE, EMBASE, CINAHL, LILACS, AMED, PsycInfo, Cochrane Library, Web of Science) from the beginning of the databases until February 20th, trial registries (clinicaltrials.gov, trialsearch.who.int, clinicaltrialsregister.eu), preprint servers (OSF, medRxiv), ProQuest Dissertations & Theses Citation Index and oatd.org for theses, as well as the Indian Ministry of AYUSH research portal (ayushportal.nic.in, google scholar). The search strategy combined terms regarding homeopathy, study design (randomized controlled trials (RCTs) and non-randomized studies of interventions (NRSIs), and insomnia, without language or search filter restrictions. The search strategy was developed by one reviewer (ML) and checked by an information specialist (SK). Details of the search strategy are documented in Supplementary File 2.

Results from the literature searches were exported to Covidence systematic review software, Veritas Health Innovation, Melbourne, Australia (available at www.covidence.org). After removal of duplicates, two reviewers (ML, SK) independently assessed study eligibility. Differences of opinion were resolved through discussion.

### 2.2 Eligibility criteria

Studies were eligible if they were prospective, comparative, and evaluated homeopathic preparations in participants with insomnia as the index condition. We included randomized controlled trials (RCTs) and prospective non-randomized studies of interventions (NRSIs) to capture both experimental and real-world evidence.

Participants had insomnia as the index condition, defined as an author-reported insomnia diagnosis or designation, or explicit insomnia symptoms that determined recruitment and were targeted by the intervention. Studies assessing sleep only as a secondary outcome in populations recruited for another condition were excluded. No uniform diagnostic standard was required, and eligibility was not restricted by insomnia subtype or reported etiology, reflecting expected variation across study periods and adult and pediatric populations. Study-specific definitions, diagnostic bases, and reported exclusion criteria are detailed in Supplementary File 7; non-reporting was recorded. All exclusions were documented.

Interventions comprised any HP prepared in accordance with a nationally recognized homeopathic pharmacopeia, administered by any route (e.g., oral, sublingual, topical, olfactory). Both individualized (classical) and non-individualized homeopathy were eligible. Co-interventions were permitted if the comparator group received an identical regimen excluding the HMP, ensuring the specific effect of homeopathy could be isolated.

Comparator arms included inactive controls (e.g., placebo, no treatment, waiting list) and active comparators (e.g., pharmacological agents, psychological therapies, or physical interventions). Studies were required to assess at least one insomnia-related clinical outcome at a minimum of two time points (pre- and post-intervention). Studies reporting only economic outcomes were excluded.

We excluded retrospective designs, cross-sectional studies, uncontrolled before–after studies, interrupted time series, case series, case reports, and any study subject to retraction or formal expression of concern. A detailed summary of the inclusion and exclusion criteria is provided in Supplementary File 3. No language restrictions or other limitations were applied. Details of studies excluded after full-text screening, including reasons for exclusion, are provided in Table S.3 in Supplementary File 4.

### 2.3 Data-extraction

Two reviewers (ML, DS) independently extracted data into Covidence using a structured codebook, describing the extracted variables. Variables included publication details (country, year), study design, participant demographics (mean age, gender, sample size, dropouts), intervention characteristics (type of homeopathy, potency, frequency), comparator type, duration of intervention, primary and secondary outcomes, effect size estimates, and funding sources. Discrepancies between reviewers were resolved through discussion. The codebook is provided in Supplementary File 5.

The study authors' definition of insomnia and the basis on which it was established were recorded as part of the detailed study characteristics (Supplementary File 7). The codebook-based extraction separately captured reported outcomes relating to insomnia severity, sleep quality, and sleep parameters (e.g., sleep-onset latency, total sleep time, and sleep efficiency). These outcome variables were not used to define insomnia.

For each study, pre–post mean differences and their corresponding standard deviations were extracted for treatment and control groups and analyzed as outcome measures. Further details on author contact are provided in Supplementary File 4.

### 2.4 Risk of bias

To assess the risk of bias, the Cochrane risk-of-bias tool for randomized trials (RoB 2) was used for RCTs and its supplement for crossover trials (Sterne et al., 2019) and Cochrane's ROBINS-I V2 for NRSIs (Sterne et al., 2025). Assessments followed the standard domains and decision algorithms as specified in the respective tools. Studies were evaluated on an outcome-specific basis by two reviewers independently (ML, DS), and disagreements were resolved through discussion.

### 2.5 Further assessments

Model validity of RCTs was assessed using the MVHT instrument (overall judgement: inadequate, uncertain, acceptable) (Mathie et al., 2012). Two experienced physicians and homeopaths (AD, DW) rated all studies independently; disagreements were resolved by discussion mediated by a third reviewer (ML). Model validity complements conventional risk-of-bias assessment by examining whether the intervention constituted a fair test of homeopathy. A trial may therefore have low risk of bias but limited interpretability if model validity is poor (Mathie et al., 2012).

To position trials on the efficacy–effectiveness continuum, we applied the RITES tool (scale from 1 to 5, with 1 = strong emphasis on efficacy and 5 = strong emphasis on effectiveness) (Wieland et al., 2017). The same reviewers (AD, DW) performed independent ratings; disagreements were resolved by discussion mediated by a third reviewer (ML). We applied a modified version of the Intervention Complexity Assessment Tool for Systematic Reviews (iCAT_SR), adapted to homeopathy-relevant dimensions, including the degree of individualization, consultation characteristics, and integration with co-interventions (Lewin et al., 2016). Two reviewers (DW, ML) independently coded eligible studies according to these dimensions, with disagreements resolved by consensus. The resulting profiles were summarized descriptively.

### 2.6 Statistical analysis

Data synthesis was guided by the Intervention Synthesis Questions (InSynQ) framework (Brennan et al., 2023). The corresponding checklist and synthesis plan are provided in Supplementary File 6.

Mean differences (MDs) were reported in their natural scale direction to preserve the interpretability of the original instruments (e.g., higher scores indicating worse outcomes). When necessary to synthesize outcomes across different scales, standardized mean differences (SMDs) were computed using Hedges' g, following the standard formula


$$\begin{array}{l} g=J^{*}\frac{\bar{X_{1}}-\bar{X_{2}}}{s_{p}} \end{array}$$ (1)

where J is the small-sample correction factor and s_p_ is the pooled standard deviation (Hedges and Olkin, 1985). Effect directions were standardized such that positive values (>0) reflect a beneficial intervention effect.

If multiple time points were reported, data from the longest follow-up were extracted. Missing standard deviations for change-from-baseline values were imputed according to the Cochrane Handbook (Section 6.5.2.8), assuming a correlation coefficient of 0.5 as recommended by Fu et al. (Fu et al., 2013). When required information was not directly reported, additional statistics were derived where possible from other reported information (e.g., confidence intervals, *p*-values, test statistics) following (Higgins et al., 2024). Where necessary, effect estimates were aligned to a consistent direction across studies; odds ratios were converted to standardized mean differences using the method described by (Hasselblad and Hedges, 1995) (see Supplementary File 4). Sensitivity analyses were conducted where approximations were necessary.

For symptomatic outcomes, RCTs and NRSIs were synthesized separately. HP was compared independently against placebo and active controls. Analyses were also stratified by age group, with adults and children analyzed separately. For adverse events, RCTs and NRSIs were analyzed together using a narrative approach.

We conducted random-effects meta-analyses using the Paule-Mandel estimator for between-study variance τ2 (Harrer et al., 2021), with confidence intervals calculated using the Knapp-Hartung adjustment (KNHA) (Inthout et al., 2014). Between-study heterogeneity was evaluated using Cochrane's *Q-*test and quantified with the I^2^ statistic (Higgins and Thompson, 2002) as well as a prediction interval (Inthout et al., 2016). According to commonly used benchmarks, *I*^2^ values in the range of 30%−60% suggest moderate heterogeneity, 50%−90% indicate substantial heterogeneity, and 75%−100% reflect considerable heterogeneity (Higgins et al., 2023).

As an exploratory analysis, we calculated the overall symptom reduction to estimate the effect of HPs on insomnia across different studies with varying outcomes. To account for potential dependencies among effect sizes originating from the same study, we employed a multilevel meta-analytic approach. This model considers sampling variance at the level of individual effects (level 1), variance within studies (level 2), and variance between studies (level 3) (Cheung, 2014; Assink and Wibbelink, 2016; Harrer et al., 2021; Gucciardi et al., 2022). The analysis was conducted using the rma.mv function from the metafor package in R, assuming an intra-study correlation (rho) of 0.5 and incorporating the HKNA for aggregated data (Knapp and Hartung, 2003).

To test for possible publication bias, we visually controlled for funnel plot asymmetry, but did not conduct statistical tests due to the low number of studies (Sterne et al., 2011).

We conducted sensitivity analyses to assess the robustness of our findings and to examine the impact of alternative assumptions or methods:

- Sleep Quality: We included the within-subject crossover study by Bell et al. (2011).
- Total Sleep Duration: We assessed the effect of (a) excluding studies with data imputations as specified in Supplementary File 4.1, (b) including the Bell et al. (2011) study, and (c) including only studies with low or moderate risk of bias.
- Overall Symptom Reduction: We examined the impact of varying the intracluster correlation coefficient (rho), comparing values of rho = 0, rho = 0.5, and rho = 0.9.

The meta-analyses were conducted using R 4.3.3 (R. Core Team, 2024) and the packages metafor 4.8-0, meta 7.0-0, and bayesmeta 3.4.

### 2.7 Certainty of evidence

We assessed the certainty of evidence using the GRADE approach (Hultcrantz et al., 2017) and rated the certainty in a non-null effect. Ratings of certainty started from “high certainty” for both RCTs and NRSIs, as the later were assessed with ROBINS-I (Schünemann et al., 2019), and included consideration of risk of bias, inconsistency, indirectness, imprecision, and publication bias. GRADE was assessed by two reviewers (DS, ML) with evidence synthesis background, checked by a reviewer with a medical background (AD) and also checked by a GRADE expert (LY, see acknowledgment).

### 2.8 Patient and public involvement

Patient and public involvement (PPI) was incorporated to improve the accessibility and relevance of the Plain Language Summary (PLS) accompanying this systematic review. Following guideline from the Cochrane Consumer Network and GRIPP2, a draft PLS was shared with nine stakeholders, including six individuals with lived experience of insomnia, three public and/or health-system stakeholders experienced in reviewing patient information materials. Participants provided feedback on clarity, terminology, interpretation of findings, and relevance for patients. Feedback was thematically analyzed, and revisions were made to enhance accessibility while preserving accuracy of the evidence synthesis.

## 3 Results

### 3.1 Literature search and study selection

The database search identified 1,143 records. After removing 263 duplicates and one retracted article, 876 records were excluded during title and abstract screening. Following full-text screening of 22 reports, 16 reports pertaining to 12 independent studies were included; in two cases, the same study was reported in both a doctoral dissertation and a journal article. The study selection process is detailed in the PRISMA flow diagram (Figure 1). During full-text screening, six studies were excluded; four due to heterogeneous, non–insomnia-specific study populations.

**Figure 1:**
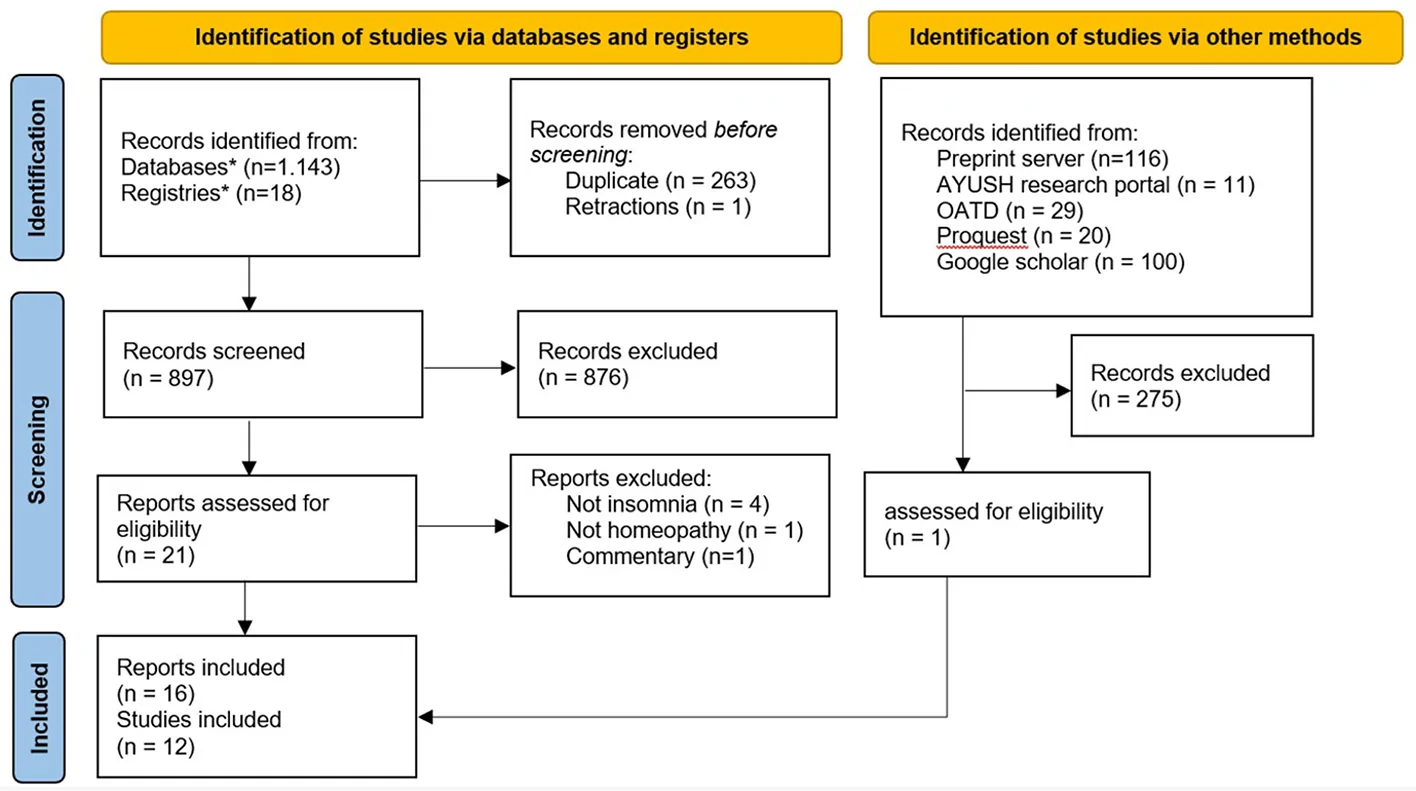
Flow diagram of study search and selection (13.8.2025). *Details: Databases: AMED (*n* = 62), CINAHL (n=225), Cochrane Library (n=103), EMBASE (*n* = 368), HOMIS (*n* = 13), Lilacs (*n* = 95), MEDLINE (*n* = 95), PsycInfo (*n* = 45), Web of Science (*n* = 137); Registries: Clinicaltrials.gov (*n* = 2), trialsearch.who.int (*n* = 7), clinicaltrialsregister.eu (*n* = 9)

### 3.2 Study characteristics

Study characteristics are presented in detail in Supplementary File 7. Eight RCTs (Carlini et al., 1987; Kolia-Adam, 2010; Naudé et al., 2010; Hejazi et al., 2012; Harrison et al., 2013; Jong et al., 2016; Michael et al., 2019; Prajakta et al., 2024) and four NRSIs (Colombo, 2003; Waldschütz and Klein, 2008; Bell et al., 2011; Prosper et al., 2016) met the eligibility criteria. Study populations comprised adults and children with insomnia as the index condition, with sample sizes ranging from 28 to 320 subjects. Definitions ranged from recognized diagnostic frameworks to study-specific symptom criteria or author-reported insomnia without a stated standard. Details are provided in Supplementary File 7. The studies comprised journal articles (Carlini et al., 1987; Waldschütz and Klein, 2008; Naudé et al., 2010; Bell et al., 2011; Harrison et al., 2013; Jong et al., 2016; Prosper et al., 2016; Michael et al., 2019; Prajakta et al., 2024), conference abstracts or proceedings (Colombo, 2003; Hejazi et al., 2012), and a thesis (Kolia-Adam, 2010). Study durations ranged from four weeks (Waldschütz and Klein, 2008; Kolia-Adam, 2010; Naudé et al., 2010; Bell et al., 2011; Hejazi et al., 2012; Harrison et al., 2013; Jong et al., 2016), 6 weeks (Prajakta et al., 2024), 3 months (Carlini et al., 1987; Colombo, 2003; Michael et al., 2019) to follow-up periods of up to 6 months (Prosper et al., 2016). Interventions included individualized homeopathic treatment (Carlini et al., 1987; Naudé et al., 2010; Prosper et al., 2016; Michael et al., 2019; Prajakta et al., 2024), specific single remedies such as *Nux vomica* or *coffea cruda* (Kolia-Adam, 2010; Bell et al., 2011; Hejazi et al., 2012), and complex homeopathic preparations (Colombo, 2003; Waldschütz and Klein, 2008; Harrison et al., 2013; Jong et al., 2016). Comparators comprised placebo controls in most randomized trials (Carlini et al., 1987; Kolia-Adam, 2010; Naudé et al., 2010; Harrison et al., 2013; Michael et al., 2019; Prajakta et al., 2024) as well as active or usual-care controls in RCTs (Hejazi et al., 2012; Jong et al., 2016) and non-randomized studies (Colombo, 2003; Waldschütz and Klein, 2008; Prosper et al., 2016). Outcomes varied substantially across studies, covering multiple domains of sleep architecture, insomnia severity, daytime functioning, psychological symptoms, and patient-reported outcomes.

### 3.3 Risk of bias

Risk of bias was assessed separately for each outcome and is reported alongside the corresponding meta-analyses (Supplementary File 8). Differences in risk of bias across outcomes within individual studies were rare. Michael et al. was the only RCT rated at low risk of bias across all assessed outcomes. Three of four NRSIs were judged to have a serious (Waldschütz and Klein, 2008) or critical risk of bias (Colombo, 2003; Prosper et al., 2016), which led to the exclusion of the two studies with critical risk of bias from the analysis of the total sleep duration (see Supplementary Figure 2d, Supplementary File 12). The predominant sources of bias were residual confounding due to inadequate adjustment, selection bias, and serious bias in outcome measurement. One RCT (Carlini et al., 1987) and one NRSI (Bell et al., 2011) were assessed using the risk-of-bias instrument for cross-over RCTs (see Figure 2c in supplementary File 8).

### 3.4 Intervention-specific assessments

Model validity, assessing the degree to which the intervention reflects adequate homeopathic practice, was acceptable in four of the RCTs (Kolia-Adam, 2010; Naudé et al., 2010; Michael et al., 2019; Prajakta et al., 2024), indicating that the intervention was sufficiently representative of standard homeopathic practice. The validity of the four remaining RCTs was classified as uncertain, largely due to insufficient reporting regarding the rationale (Domain I) or principles (Domain II) of the therapy. The position of the studies on the efficacy–effectiveness continuum was assessed using the RITES tool, revealing considerable heterogeneity in trial design and real-world applicability. The mean of the domain-specific average scores of the eight RCTs on the RITES scale (1 = efficacy-oriented, 5 = effectiveness-oriented) was *m* = 2.25 (range: 1.25–4). The details of MVHT and RITES assessment scores are provided in Supplementary File 9. The NRSIs were assessed with the instruments on an exploratory basis only.

Intervention complexity assessed with iCAT_SR varied across the studies (8 RCTs, 2 NRSIs). Half of the studies assessed single-component interventions (50%). Tailoring was most often complex or fully individualized (30% each). Practitioner skill, component interaction, and dependence on recipient or provider factors showed bimodal distributions, with equal proportions at low and high levels. The details of iCAT assessment are shown in Supplementary File 10.

### 3.5 Effects of intervention

#### 3.5.1 Homeopathy vs. placebo in adults

An overview of the results is presented in Table 1 and Certainty of Evidence is presented in Supplementary File 11.

**Table 1:** Homeopathy vs. placebo in adults for sleep-related outcomes in adults.

Outcome	Moderator	Scales	Studies	MD	95% CI		I2	Figure in supp. 8
Quality of sleep	–	Pittsburgh sleep quality index	1	−2.62 points	−5.5	0.26		1a
	INCLUSION of non-randomized study (Bell 2011)	Pittsburgh sleep quality index	2	−0.99 points	−3.21	1.23	61%	9
Total sleep duration	–	Sleep diary	3	0.65 h	−0.9	2.2	77%	2a
	Excluding studies with imputed data (Harrison 2013; Kolia-Adam 2010); =only study with low risk of bias	Sleep diary	1	0.7 h	0.42	0.98		10
	Inclusion of non-randomized study (Bell 2011)	Sleep diary, in-home ambulatory polysomnography	4	0.77 h	−0.13	1.68	76%	11
	Studies with low/moderate risk of bias	Sleep diary	2	0.4 h	−0.24	1.05	80%	12
Insomnia severity	–	Insomnia severity index	1	−3.2 points	−5.68	−0.72		3a
Sleep onset latency	–	Sleep diary	1	−5.6 min	−15.35	4.15		4a
Sleep efficiency	–	Sleep diary	1	6.9 points	3.99	9.81		5a

##### 3.5.1.1 Sleep quality

Sleep quality was informed by a single RCT (*n* = 30) (Prajakta et al., 2024); the certainty of evidence for this outcome was rated as low. In the primary analysis, individualized homeopathy did not significantly improve sleep quality compared with placebo (MD = −2.62, 95% CI −5.50 to 0.26) [Figure 1a/b in Supplementary File 8] which was supported in sensitivity analysis when including a within subject crossover NRSI [Figure 9 in Supplementary File 8].

##### 3.5.1.2 Total sleep duration

Three RCTs (Kolia-Adam, 2010; Harrison et al., 2013; Michael et al., 2019), comprising a total of 124 participants, assessed total sleep duration (hours) using sleep diaries. The primary meta-analysis demonstrated no statistically significant difference between homeopathy and placebo (MD = 0.65; 95% CI −0.9 to 2.2), [Figure 2a/b in Supplementary File 8]. The certainty of evidence was rated as low. Sensitivity analyses yielded consistent findings (see Table 1). Naudé et al. (2010) reported between-group differences in sleep diary–based sleep duration but were excluded from the quantitative synthesis due to unavailable variance estimates (see Supplementary File 4).

##### 3.5.1.3 Insomnia severity

One RCT involving 60 participants assessed the Insomnia Severity Index and was included in the quantitative synthesis (MD = −3.2; 95% CI −5.7 to −0.7) [Supplementary Figure 3a/b, Supplementary File 10]. The certainty of evidence was rated as moderate. Naudé et al. (2010) also assessed insomnia severity using the Sleep Impairment Index and reported between-group differences.

Sleep onset latency and sleep efficiency. *One* RCT (Michael et al., 2019); *n* = 60), judged at low risk of bias, assessed both outcomes. Homeopathy was associated with an MD = −5.60 min (95% CI −15.35 to 4.15; low certainty) in sleep onset latency and a improvement in sleep efficiency (MD = 6.90; 95% CI 3.99 to 9.81; moderate certainty) compared with placebo [Figures 4a/, 5a/b in Supplementary File 8].

##### 3.5.1.4 Adverse events

Only 2 of the 9 studies reported methods and results on adverse events (Waldschütz and Klein, 2008; Michael et al., 2019), [see Supplementary File 8]. In Michael et al. (3-month intervention), one mild case of rectal bleeding occurred and was managed with homeopathic treatment; no serious AEs were reported (Michael et al., 2019) [see Figure 7a in Supplementary File 8 for risk of bias]. In one NRSI (4-week intervention), a single case of mild caffeine intolerance was observed [Figure 7b in Supplementary File 8 for risk of bias]. Both studies explicitly monitored AEs and reported high tolerability (Waldschütz and Klein, 2008). The remaining studies either did not monitor or did not report AEs, limiting the ability to estimate AE frequency reliably. This outcome is assessed to have low certainty of evidence.

In an exploratory analysis, multiple sleep-related outcomes (total sleep time, insomnia severity, sleep-onset latency, sleep efficiency, and sleep quality) from five studies (Kolia-Adam, 2010; Harrison et al., 2013; Michael et al., 2019; Prajakta et al., 2024) were pooled, yielding an SMD of 0.73 (95% CI [0.04, 1.41]). [Figures 8, 13a–c in Supplementary File 8].

#### 3.5.2 Homeopathy vs. placebo in children

##### 3.5.2.1 Insomnia severity

Evidence for this outcome was derived from a single open-label randomized trial in children under 6 years with sleep disorders (*n* = 176) (Jong et al., 2016). The certainty of evidence was rated as very low. The study reported a large effect favoring homeopathy over the active comparator (glycine) in reducing insomnia severity (SMD: 0.82, 95% CI 0.56 to 1.09) [Figure 6a/b in Supplementary File 8] which corresponds to a reported odds ratio of 4.45 (95% CI 2.77 to 7.14).

##### 3.5.2.2 Adverse events

In the same trial by Jong et al., (Jong et al., 2016), adverse events were systematically monitored (low certainty of evidence). Eleven non-serious adverse events were reported (six in the homeopathy group and five in the comparator group), most of which were considered unrelated to treatment. One child in the homeopathy group discontinued due to excitability. No serious adverse events were observed [see Figure 7c in Supplementary File 8 for risk of bias].

### 3.6 Reporting biases

The risk of bias due to missing results was assessed by visual inspection of funnel plots, however, given the small number of studies, funnel plots provide limited information and cannot be reliably interpreted, so no firm conclusions can be drawn. Formal tests for funnel plot asymmetry were not performed due to the small number of studies [Figures 14, 15 in Supplementary File 8].

### 3.7 Certainty of the evidence

For adults, evidence for adverse events, sleep quality, total sleep duration, and sleep onset latency was rated as low certainty, primarily due to high risk of bias (missing data, inadequate analyses) and imprecision (see Supplementary File 11). Evidence for insomnia severity and sleep efficiency reached moderate certainty.

For children, evidence for insomnia severity was of very low certainty and limited to a single open-label, industry-funded trial using a non-validated, proxy-reported measure. Evidence for adverse events in children was of low certainty (see Supplementary File 12).

### 3.8 Impact of patient and public involvement

Patient and public reviewers primarily suggested improvements to clarity and presentation. Explanatory phrases were added for methodological terms such as randomized and non-randomized studies, and a brief neutral description of homeopathy was included.

Reviewers also recommended presenting the review's main message earlier, acknowledging patient experiences of insomnia, and clarifying that different types of homeopathic interventions were evaluated. The section on adverse events was slightly expanded to emphasize safety considerations, and the glossary was referenced earlier in the text to improve navigation. The final version of the Plain Language Summary is presented at Supplementary File 13.

## 4 Discussion

### 4.1 Summary of findings

This systematic review assessed the evidence regarding the efficacy, effectiveness, and safety of homeopathic interventions for insomnia. Overall, the available evidence did not provide clear or consistent evidence of robust effects and was associated with uncertainty, driven by small sample sizes, high risk of bias and heterogeneity in study designs, reporting, patient populations, interventions, and outcomes. Notably, several trials were judged to reflect adequate homeopathic practice and covered a broad range along the efficacy-effectiveness continuum, indicating substantial heterogeneity in intervention complexity and real-world applicability.

In adults, placebo-controlled evidence was inconclusive with respect to consistent benefits of homeopathy for core sleep outcomes. Signals of potential improvement in sleep duration emerged but were not statistically significant. Evidence for insomnia severity and other sleep parameters was limited to single studies, yielding low to moderate certainty.

In children, evidence was restricted to one open-label RCT reporting large improvements compared with an active comparator; however, very low certainty of evidence and important design limitations preclude firm conclusions.

Reporting of adverse events was sparse across studies, precluding firm conclusions regarding safety. Although the reported data did not indicate major tolerability concerns, the certainty of evidence regarding adverse events was rated as low to very low.

### 4.2 Interpretation in the context of other evidence

Taken together, our findings do not contradict earlier systematic reviews, which described the evidence for homeopathic interventions in insomnia as limited and inconclusive (Cooper and Relton, 2010; Ernst, 2011). Although the magnitude observed in our exploratory, non–outcome-specific analysis was numerically similar to that reported by (Paracha et al., 2026), direct comparison is limited by differences in search scope, study selection, comparators, and analytical approach. Our estimate was substantially driven by a single study contributing multiple outcomes and around 50% of the meta-analytic weight.

A complex-systems perspective complements primary-endpoint analysis by considering whether interdependent changes across multiple outcome domains reflect a broader response in the patient as a whole (Van Haselen et al., 2026). However, our exploratory pooling of heterogeneous sleep outcomes cannot establish such a response, and the current evidence is insufficient for this interpretation.

Adverse events were infrequent and non-serious, consistent with findings from observational studies (Stub et al., 2020). In that review, however, adverse events were systematically monitored and comprehensively reported in most included studies. By contrast, only a minority of trials in the present analysis reported active monitoring of adverse events. Due to a likely absence of reporting, estimates of adverse event frequency remain imprecise.

Our assessment of the certainty of evidence was anchored to the null threshold, focusing on the presence of a relative effect rather than its magnitude, in line with established conceptual guidance (Hultcrantz et al., 2017) and considerations for complex interventions (Montgomery et al., 2019). In parallel, and consistent with recent GRADE guidance (Zeng et al., 2025), we examined whether observed point estimates were smaller than the smallest plausible minimal important differences (MIDs) to evaluate whether a target of little or no effect would be more appropriate.

For several outcomes, including total sleep time and sleep onset latency, the absence of established MID values precluded application of this approach. For sleep quality, the observed effect exceeded the proposed MID of 2.5 points (Qin et al., 2024) and the non-null target was retained. For insomnia severity, published thresholds are heterogeneous and population-specific, and include estimates derived from different instruments or patient populations (Yang et al., 2009; Lenderking et al., 2024). Given these limitations, no definitive MID-based target modification was applied. A MID for total sleep duration has recently been proposed (Phillips-Beyer et al., 2024); however, its applicability to the present evidence base remains uncertain given the heterogeneity across studies. For sleep onset latency and sleep efficiency, interpretation was further constrained by the lack of established MID thresholds.

Against this background, the effects observed for homeopathic interventions should be interpreted with caution. The available evidence is constrained by imprecision, heterogeneity, small study numbers, and limited adverse-event reporting, which together preclude firm conclusions regarding both effectiveness and safety. This pattern of findings aligns with current insomnia guidelines, which consistently recommend cognitive behavioral therapy for insomnia (CBT-I) as first-line treatment, while reserving pharmacological therapies for selected patients and typically for short-term use (Qaseem et al., 2016; Riemann et al., 2017).

The preferential position of CBT-I in clinical guidelines is supported by a large and coherent body of evidence demonstrating clinically meaningful and durable improvements in insomnia severity and key sleep parameters (Trauer et al., 2015). Pharmacological treatments, by contrast, may offer short-term benefits, as shown in network meta-analyses, but require careful benefit–risk assessment (Yue et al., 2023); concerns are particularly pronounced for Z-drugs in older adults, where associations with falls and fractures have been reported (Scharner et al., 2022). More broadly, the adverse-effect burden of pharmacological insomnia treatments is a clinically relevant reason to prioritize non-pharmacological strategies whenever feasible.

At the same time, CBT-I remains underutilized in routine care due to barriers in access, availability, and implementation (Koffel et al., 2018). In this context, patient interest in low-threshold and perceived low-risk complementary approaches remains understandable, particularly where patients wish to avoid or minimize exposure to pharmacological treatment (Cheung et al., 2018; Sato et al., 2019; Perez et al., 2022). However, the current evidence does not allow homeopathy to be positioned as an evidence-based alternative or adjunct in insomnia care.

Interpreting the findings of this review through a complexity-informed lens provides additional context for understanding the pronounced heterogeneity observed across studies. Homeopathy, as examined in the included trials, does not constitute a single, uniform intervention but rather encompasses approaches that differ with respect to treatment individualization, prescribing strategies, and trial design.

A modified iCAT was applied with the aim of supporting a more structured reflection on intervention complexity within the evidence base (Lewin et al., 2017); however, limited reporting in the primary studies rarely allowed a differentiated assessment across multiple dimensions. Consequently, its application was largely restricted to a high-level characterization, most notably with respect to treatment individualization, illustrating the challenges of operationalizing complexity-oriented frameworks when key contextual and process-related information is insufficiently reported.

Complementary assessments of model validity and trial pragmatism further highlighted the heterogeneity of the evidence base. According to the MVHT assessment, fewer than half of the included studies demonstrated acceptable concordance with contemporary homeopathic practice, while the RITES tool indicated a broad spectrum of designs ranging from more explanatory trials to studies with more pragmatic features. Together, these findings indicate that variability in intervention characteristics and study design is a key source of uncertainty in effect estimation and interpretation.

### 4.3 Strengths and limitations of the evidence

The results of this review should be interpreted in light of general limitations of the available evidence base. The included studies were predominantly small, frequently exhibited an elevated risk of bias, and showed substantial heterogeneity, including variability in reported outcomes. Several studies were identified as gray literature and demonstrated shortcomings in reporting quality. Only three studies defined their populations according to diagnostic reference standards (see Supplementary File 7). Because no uniform diagnostic standard was required and reporting was often incomplete, diagnostic specificity and the exclusion of alternative causes varied across studies. This limits clinical comparability and subtype-specific interpretation.

The broad inclusion strategy of this review, which encompassed all prospective homeopathy RCTs and NRSIs in insomnia disorder, reflects the characteristics of the existing research field but also entails considerable clinical heterogeneity. This is particularly relevant given the joint consideration of individualized homeopathy and complex preparations and may have contributed to the observed statistical heterogeneity. Even if diagnostic procedures had been more consistently reported, the small number of available studies precluded moderator analyses to assess the influence of these or other factors on effect estimates. Sleep outcomes were predominantly based on participant- or caregiver-reported rating scales and sleep diaries. Bell et al. (2011) was the only study using wrist actigraphy and ambulatory in-home polysomnography with electroencephalographic and electromyographic recordings. As this study was included only in sensitivity analyses, objective corroboration of the main findings remained limited.

Another limitation is that effectiveness under routine care conditions could not be determined due to the lack of reliable NRSIs. Two of the four identified NRSIs were judged to be at critical risk of bias and were therefore excluded from further analyses. The remaining NRSIs comprised one study at serious risk of bias and one non-randomized cross-over study included only in sensitivity analyses, precluding reliable conclusions regarding real-world effectiveness.

In addition, it was not possible to analyze global measures of symptom improvement or patient satisfaction based on the reported data, outcomes that are of particular relevance for holistic medical approaches such as homeopathy. The exploratory analysis intended to approximate overall symptom reduction by pooling different outcomes is limited, as it combines different constructs and measurement scales (Murad et al., 2019) and, in this review, is driven to a substantial extent by a single pilot study.

The evidence was further limited by methodological weaknesses in most trials, restricted safety reporting, and scarcity of pediatric data, all of which reduce confidence in the estimates and limit generalizability.

One strength of this review is the comprehensive literature search and non-restrictive inclusion criteria, which reduced the risk of missing relevant studies, including those not published in peer-reviewed journals. All stages of the review were conducted independently by two reviewers. Domain experts were involved in the MVHT and RITES assessments as well as in the clinical evaluation. Patients were involved in the development of both the protocol and the manuscript, and patient perspectives were considered in relation to clinical relevance.

Although the protocol initially prioritized the CATHIS tool, insufficient inter-rater reliability identified in a parallel validation study (Loef et al., 2026) necessitated an adapted methodological approach. The use of MVHT and RITES instead allowed a more reliable characterization of intervention fidelity and trial pragmatism based on the available reporting.

Finally, PPI improved the accessibility of the review outputs by identifying areas where technical language could limit understanding for non-specialist readers. Feedback also highlighted the importance of clearly communicating uncertainty and safety, which are key concerns for patients considering complementary treatments. Although PPI focused on improving knowledge translation rather than influencing analytical stages of the review, it helped produce a clearer and more accessible summary of the evidence.

### 4.4 Implications and conclusions

Overall, the available evidence for homeopathic treatments for insomnia is limited, and the effects remain uncertain, with no reliable evidence demonstrating a specific therapeutic effect. The existing literature is dominated by small and exploratory studies, many of which exhibit limitations in design and reporting. These shortcomings align with previously documented challenges in internal, external, and model validity within homeopathic clinical research, some of which may be attributable to incomplete or inconsistent reporting rather than study conduct *per se* (Herman et al., 2025).

From a clinical perspective, the current evidence does not provide consistent or high-certainty support for the use of homeopathy as a treatment for insomnia or general sleep disturbances in adults. While certain analyses, particularly for insomnia severity and sleep efficiency, suggest potential benefits, these signals are sensitive to methodological decisions, heterogeneity, and the inclusion of lower-quality studies. Clinicians should therefore exercise caution when considering homeopathy for insomnia and prioritize evidence-based interventions with stronger and more reliable therapeutic profiles. In pediatric populations, where only one open-label trial was available, the very low certainty of evidence and methodological limitations preclude us from providing any clinical recommendation.

In this context, additional small or methodologically limited studies are unlikely to substantially advance the evidence base and may continue to contribute only marginally to knowledge accumulation. Future research efforts should therefore focus on a rigorously designed, independently conducted confirmatory replication based on the protocol of Michael et al., which currently represents the only study within the evidence base demonstrating both low risk of bias and acceptable homeopathic model validity. Such a trial should be prospectively registered, strictly adhere to CONSORT reporting standards, and ensure full transparency and reproducibility. In addition to placebo-controlled designs, future studies may also consider comparative effectiveness designs evaluating the incremental benefit of homeopathic treatment as an adjunct to established standard care, such as cognitive behavioral therapy for insomnia.

In line with contemporary frameworks for evidence synthesis of complex and whole medical systems, which emphasize the role of context and complexity, future trials should also systematically capture and report relevant contextual factors, including consultation characteristics, patient expectations and prior experiences with homeopathy, as well as global and patient-reported outcomes such as overall satisfaction (Van Haselen et al., 2026). These elements are essential for the adequate evaluation and interpretation of homeopathy as a complex intervention. In addition, trials must incorporate active and systematic surveillance for adverse events to allow a robust assessment of safety.

Future trials should also distinguish among the clinical presentations of insomnia: difficulty with sleep initiation, difficulty with sleep maintenance, and early-morning awakening, and report outcomes accordingly. Explicitly reporting remedy selection in relation to insomnia presentation would allow a more informative, hypothesis-driven evaluation than pooling heterogeneous presentations under a single ‘insomnia' label.

From a policy and research governance perspective, these findings support greater methodological standardization, including core outcome sets, trial preregistration, and transparent safety reporting.

At the same time, given the current uncertainty of the evidence, policy decisions regarding the inclusion or exclusion of homeopathy-based interventions for insomnia in public health programs or insurance coverage would benefit from a cautious and proportionate approach. Any future investment in this field should prioritize well-powered, placebo-controlled trials with standardized outcomes and comprehensive adverse-event reporting.

Accordingly, in the absence of reproducible evidence meeting these methodological and reporting standards, the specific therapeutic value of homeopathy for insomnia remains uncertain.

## Data Availability

The datasets presented in this study can be found in online repositories. The names of the repository/repositories and accession number(s) can be found in the article/Supplementary material.

## Supplementary material

Supplementary File 1 HOM-A template protocol, with insomnia-specific adaptations.

Supplementary File 2 Literature search strategy for different databases.

Supplementary File 3 Study eligibility criteria.

Supplementary File 4 Details of studies excluded after full-text screening, including reasons for exclusion, and data handling decisions.

Supplementary File 5 Codebook.

Supplementary File 6 Intervention synthesis questions (InSynQ) framework checklist and synthesis plan.

Supplementary File 7 Study characteristics data.

Supplementary File 8 Risk of bias and meta-analysis.

Supplementary File 9 MVHT and RITES assessments.

Supplementary File 10 Details of iCAT assessment.

Supplementary File 11 Certainty of evidence for outcomes in adults.

Supplementary File 12 Certainty of evidence for outcomes in children.

Supplementary File 13 Plain language summary.
