# Supplementary material for: Homeopathy for the treatment of insomnia: a systematic review and meta-analysis
Source: Front Sleep. 2026 Sep 28;5:1922807. doi: 10.3389/frsle.2026.1922807 (PMC13654833; doi:10.3389/frsle.2026.1922807)
Supplement: Supplementary file 1 [file Data_Sheet_1.docx]

**Supplement 1**

**Purpose**
This review follows the HOMA Template Protocol V1.2 (Loef et al., 2025) in its entirety, except for adaptations necessary for the insomnia context. The table below lists each adaptation by section, with rationale.

| **Template Protocol Section** | **Adopted Without Change** | **Details of adaptation** | **Rationale** |
| --- | --- | --- | --- |
| Review team & roles | Yes |  | Same team composition and roles as in the template; no methodological change. |
| Participants | No | Adults (≥18 y) with insomnia disorder per ICSD-3; exclusion of certain comorbidities (e.g., severe psychiatric disorders), secondary insomnia, and specific medication use. | Aligns eligibility criteria with insomnia-specific diagnostic guidelines and removes populations that could confound outcomes. |
| Interventions | Yes |  | Definition of homeopathic preparations and inclusion rules identical to template. |
| Comparators | No | Explicit comparator categories: inactive controls (placebo, no treatment, wait-list) and active comparators (pharmacological agents, cognitive-behavioural therapy, other psychological/behavioural therapies). | Reflects standard insomnia treatment comparators. |
| Outcomes | No | Primary: validated sleep quality/latency/duration measures (e.g., PSQI, ISI), adverse events; Secondary: sleep efficiency, total sleep time, daytime functioning, quality of life. | Tailored to insomnia-specific clinical and patient-relevant outcomes. |
| Databases searched | No | Added PsycINFO, Sleep-related specialty databases; removed template topic-specific databases unrelated to insomnia. | Ensure coverage of insomnia and sleep research literature while focusing searches. |
| Search strategy | No | Insomnia-specific MeSH/keywords adapted from Cochrane insomnia and sleep disorder reviews; detailed executed strategies in Supplement 2. | Ensures sensitivity/specificity for insomnia literature. |
| Data extraction | Yes |  | Same as template: structured forms/codebook, dual independent extraction. |
| Risk of bias | Yes |  | Same as template |
| Validity assessment | Yes |  | Same as template |
| Data synthesis | No | Specified sensitivity analysis: Bayesian meta-analysis for small-study settings | Address small study numbers and unreliable behaviour of Hartung-Knapp adjustment with very few studies. |
| Other | No | Living review with update plan on homeopathy-evidence.info. | Ongoing surveillance for new trials relevant to insomnia. |
