# Supplementary material for: Homeopathy for the treatment of insomnia: a systematic review and meta-analysis
Source: Front Sleep. 2026 Sep 28;5:1922807. doi: 10.3389/frsle.2026.1922807 (PMC13654833; doi:10.3389/frsle.2026.1922807)
Supplement: Supplementary file 2 [file Data_Sheet_11.docx]

**Supplement 11**

**Certainty of Evidence for Outcomes in Adults**

| Outcomes | **Relative effect (95% CI)** | № of participants (studies) | Certainty of the evidence (GRADE) | Comments |
| --- | --- | --- | --- | --- |
| Sleep quality assessed with: PSQI Scale from: 0 to 21 follow-up: 6 weeks | MD **2.62 points lower** (-5.5 lower to 0.26 higher) | 30 (1 RCT) | ⨁⨁◯◯ Low^a,b^ |  |

| Sleep efficiency assessed with: sleep diary follow-up: 12 weeks | MD **6.9 points higher** (3.99 higher to 9.81 higher) | 60 (1 RCT) | ⨁⨁⨁◯ Moderate^c^ |  |
| --- | --- | --- | --- | --- |

| Total sleep duration assessed with: sleep diary follow-up: range 4 weeks to 12 weeks | MD **0.65 hours more** (0.9 fewer to 2.2 more) | 124 (3 RCTs) | ⨁⨁◯◯ Low^a,b^ |  |
| --- | --- | --- | --- | --- |
| Insomnia severity assessed with: insomnia severity index follow-up: range 4 weeks to 12 weeks | MD **3.2 points lower** (5.68 lower to 0.72 lower) | 60 (1 RCT) | ⨁⨁⨁◯ Moderate^c^ |  |
| Sleep onset latency assessed with: sleep diary follow-up: 12 weeks | MD **5.6 minutes lower** (-15.35 lower to 4.15 higher) | 60 (1 RCT) | ⨁⨁◯◯ Low^e^ |  |
| Adverse events follow-up: range 4 weeks to 12 weeks | Only 2 of the 9 studies reported on adverse events (Michael et al., 2019; Waldschütz & Klein, 2008). In *Michael et al.* (3-month intervention), one mild case of rectal bleeding occurred and was managed with homeopathic treatment; no serious AEs were reported. In *Waldschütz & Klein* (4-week intervention), a single case of mild caffeine intolerance was observed. Both studies explicitly monitored AEs and reported high tolerability.The remaining studies either did not monitor or did not report AEs, limiting the ability to estimate AE frequency reliably. | 60 (1 RCT),  320  (1 NRSI) | ⨁⨁◯◯ Low^f,g^ |  |

#### Explanations

a. Downgraded one level with regard to high risk of bias due to missing outcome data and inadequate analysis.

b. Downgraded one level for imprecision due to confidence interval including the null effect.

c. Downgraded one level for imprecision — total sample size (N≈60) well below the optimal information size, but the 95 % CI lies entirely on the beneficial side, indicating a consistent direction of effect.

d. Downgraded one level for imprecision due to low number of patients and the confidence interval crossing the null-threshold in sensitivity analyses.

e. Downgraded two levels for very serious imprecision — confidence interval crosses the null, total sample size very small (N≈60), and no minimally important difference (MID) available.

f. Downgraded one level for high risk of bias. One of two studies that report adverse events (AE) have a high risk of bias; the lack of reporting in studies that have not reported AEs introduces risk of selective outcome reporting.

g. Downgraded one level for imprecision. Two out of 9 studies reported AEs. Most reported events were few and non-serious. The total number of events is very small, making it difficult to estimate the true incidence of AEs with precision.
