# Supplementary material for: Homeopathy for the treatment of insomnia: a systematic review and meta-analysis
Source: Front Sleep. 2026 Sep 28;5:1922807. doi: 10.3389/frsle.2026.1922807 (PMC13654833; doi:10.3389/frsle.2026.1922807)
Supplement: Supplementary file 3 [file Data_Sheet_12.docx]

**Supplement 11**

**Certainty of Evidence for Outcomes in Children**

**Summary of findings:**

**Homeopathy compared to active comparators for insomnia in children**

**Patient or population:** insomnia in children

**Setting:** outpatient

**Intervention:** homeopathy

**Comparison:** active comparators

| Outcomes | **Relative effect (95% CI)** | | № of participants (studies) | | Certainty of the evidence (GRADE) | Comments | |
| --- | --- | --- | --- | --- | --- | --- | --- |
| Insomnia severity follow-up: 4 weeks | SMD **0.82 SD higher** (0.56 higher to 1.09 higher) | | 176 (1 RCT) | | ⨁◯◯◯ Very low^a,b,c^ |  | |
| Adverse events follow-up: 4 weeks | | In this randomized controlled trial involving 179 children under 6 years with sleep disorders, adverse events were systematically monitored. A total of 11 non-serious events were reported (6 in the homeopathy group, 5 in the comparator group), most unrelated to treatment. One child in the homeopathy group withdrew due to excitability. No serious adverse events occurred. While the trial was open-label, AE monitoring appeared unbiased. | | 176 (1 RCT) | ⨁⨁◯◯ Low^c,d^ | |  |

***The risk in the intervention group** (and its 95% confidence interval) is based on the assumed risk in the comparison group and the **relative effect** of the intervention (and its 95% CI).

**CI:** confidence interval; **SMD:** standardised mean difference

**GRADE Working Group grades of evidence**
**High certainty:** we are very confident that the true effect lies close to that of the estimate of the effect.
**Moderate certainty:** we are moderately confident in the effect estimate: the true effect is likely to be close to the estimate of the effect, but there is a possibility that it is substantially different.
**Low certainty:** our confidence in the effect estimate is limited: the true effect may be substantially different from the estimate of the effect.
**Very low certainty:** we have very little confidence in the effect estimate: the true effect is likely to be substantially different from the estimate of effect.

#### Explanations

a. Downgraded one level for serious risk of bias due to missing blinding and subjective outcome measures.

b. Downgraded one level for indirectness due to use of non-validated, proxy-reported composite measure of sleep disturbance severity.

c. Downgraded one level for publication bias. The only available study is industry-funded. Given the absence of independent studies and the potential for selective non-publication, we downgraded the certainty of evidence by one level for suspected publication bias.

d. Only 11 adverse events occurred in total (6 in the homeopathy group), all non-serious and mostly unrelated. The small number of events and the limited sample (N=179) make it difficult to precisely estimate risk.
