# Supplementary material for: Homeopathy for the treatment of insomnia: a systematic review and meta-analysis
Source: Front Sleep. 2026 Sep 28;5:1922807. doi: 10.3389/frsle.2026.1922807 (PMC13654833; doi:10.3389/frsle.2026.1922807)
Supplement: Supplementary file 4 [file Data_Sheet_13.docx]

**Supplement 13**

**Plain Language Summary**

**What is insomnia?**

Insomnia is a common sleep condition. People with insomnia may have difficulty falling asleep, staying asleep, or may wake too early and feel unrefreshed. Ongoing sleep problems can affect mood, concentration, daily activities, and overall quality of life. Many people with insomnia try different approaches to improve their sleep.

Key sleep-related terms used in this summary are explained in the glossary at the end.

**Why was this review done?**

Homeopathy is a system of treatment that uses highly diluted (and potentised) substances chosen to match a person’s symptoms. Some people use homeopathy as a complementary approach for insomnia. However, it is unclear whether homeopathic treatments improve sleep or whether they are safe. This review examined the available research evidence on homeopathy for insomnia in adults and children.

**What did the researchers do?**

The review identified and analysed studies that compared homeopathic treatments with placebo (a dummy treatment) or other comparison treatments. Only studies that followed people forward in time were included. The review assessed effects on sleep outcomes and looked for reports of unwanted or harmful effects (side effects). The reliability of the studies was also carefully evaluated.

**What studies were found?**

Twelve studies were included:

- Eight randomized controlled trials, where participants were randomly assigned to different treatments to allow fair comparison.
- Four non-randomized studies, where treatments were compared but participants were not randomly assigned.

The studies differed in how insomnia was defined, the types of homeopathic treatments used (including individualized remedies and standardized preparations), how long treatment lasted, and which sleep outcomes were measured. These differences make the findings difficult to compare and interpret.

**What were the main findings?**

*Adults*

- One study found that homeopathic treatment may reduce the severity of insomnia symptoms. This suggests a possible benefit, but the finding comes from a single study.
- For sleep quality and total sleep time, the results did not show a clear difference between homeopathy and placebo.
- One study reported better sleep efficiency with homeopathy, while no clear effect was seen for time taken to fall asleep. These findings should be interpreted cautiously because they were based on individual studies.

*Children*

- Only one study in children assessed insomnia severity. It suggested a difference in favour of homeopathy, but the evidence was very uncertain due to major study limitations.

**Side effects**

Few studies actively monitored side effects. Mild problems were reported in a small number of participants, and no serious harms were described. However, because most studies did not report side effects, there is not enough reliable information to draw clear conclusions about safety.

**How reliable is the evidence?**

Overall, the evidence is limited. Many studies included small numbers of participants and had weaknesses in their design or reporting. Findings were often based on single studies and were not consistently repeated. This means that results could change if better-quality studies are conducted.

**What does this mean?**

Current evidence does not provide clear answers about whether homeopathic treatments help people with insomnia. While some findings suggest a possible benefit for certain outcomes, the evidence is uncertain and incomplete. Better-designed and larger studies, with careful reporting of side effects, are needed to clarify the role of homeopathy in insomnia.

**Glossary of terms:**

- **Sleep quality**: how well people felt they slept.
- **Total sleep time**: how many hours people slept per night.
- **Insomnia severity**: how severe sleep problems were, usually measured with questionnaires about sleep difficulties and their impact.
- **Time to fall asleep (sleep onset latency)**: how long it took to fall asleep.
- **Sleep efficiency**: how much of the time spent in bed was actually spent sleeping.
- **Adverse events**: unwanted or harmful effects during treatment.
