# Supplementary material for: Homeopathy for the treatment of insomnia: a systematic review and meta-analysis
Source: Front Sleep. 2026 Sep 28;5:1922807. doi: 10.3389/frsle.2026.1922807 (PMC13654833; doi:10.3389/frsle.2026.1922807)
Supplement: Supplementary file 5 [file Data_Sheet_2.docx]

**Supplement 2**

# Literature Search Strategy for Different Databases

## MEDLINE (OVID)

homeopathy

#1 Homeopathy/ or Materia Medica/ or Pharmacopoeias, Homeopathic as Topic/ or Formularies, Homeopathic as Topic/ or (homeopath* or homoeopath* or homoop* or omeop* or homopath* or potentis* or potentiz* or "materia medica" or nosode*).tw.

study type

*randomized controlled trials*

#2 (exp Randomized Controlled Trial/ OR "controlled clinical trial".pt. OR randomized.ab. OR placebo.ab. OR "Drug Therapy".fs. OR randomly.ab. OR trial.ab. OR groups.ab.) NOT (exp animals/ NOT humans.sh.)

*non-randomized studies on interventions*

#3 (exp cohort studies/or exp epidemiologic studies/or exp clinical trial/or exp evaluation studies as topic/or exp statistics as topic/ or ((control and (group* or study)) or (time and factors) or program or survey* or ci or cohort or comparative stud* or evaluation studies or follow-up*).mp.) not ((animals/not humans/) or comment/or editorial/or exp review/or meta analysis/or consensus/or exp guideline/ or hi.fs. or case report.mp.)

condition (e.g. insomnia)

#4 exp "Sleep Initiation and Maintenance Disorders"/ or insomn*.ab,ti. OR sleep*.ab,ti. OR dyssomn*.ab,ti

#5	#2 OR #3

#6	#1 AND #4 AND #5

## EMBASE (OVID)

homeopathy

#1 homeopathic agent/ or homeopathy/ or materia medica/ or (homeopath* or homoeopath* or homoop* or omeop* or homopath* or potentis* or potentiz* or "materia medica" or nosode*).tw.

study type

*randomized controlled trials*

#2 (exp randomized controlled trial/ or controlled clinical trial/ or random$.ti,ab. or randomization/ or intermethod comparison/ or placebo.ti,ab. or (compare OR compared OR comparison).ti,ab. or  ((evaluated OR evaluate OR evaluating OR assessed OR assess) AND (compare OR compared OR comparing OR comparison)).ab or (open adj label).ti,ab. or ((double OR single OR doubly OR singly) adj (blind OR blinded OR blindly)).ti,ab. or double blind procedure/ or parallel group$1.ti,ab. or (crossover OR cross over).ti,ab. or ((assign$ OR match OR matched OR allocation) adj5 (alternate OR group$1 OR intervention$1 OR patient$1 OR subject$1 OR participant$1)).ti,ab. or (assigned OR allocated).ti,ab. or (controlled adj7 (study OR design OR trial)).ti,ab. or (volunteer OR volunteers).ti,ab. or human experiment/ or trial.ti.) not ((random$ adj sampl$ adj7 ("cross section$" OR questionnaire$1 OR survey$ OR database$1)).ti,ab. NOT (comparative study/ OR controlled study/ OR randomi?ed controlled.ti,ab. OR randomly assigned.ti,ab.) or cross-sectional study/ NOT (exp randomized controlled trial/ OR controlled clinical trial/ OR controlled study/ OR randomi?ed controlled.ti,ab. OR control group$1.ti,ab.) or ((case adj control$) AND random$.ti,ab.) NOT randomi?ed controlled.ti,ab. or (systematic review.ti,ab. NOT (trial OR study).ti) or    (non random$ NOT random$).ti,ab. or "random field$".ti,ab. or (random cluster adj3 sample$).ti,ab. or (review.ab. AND review.pt.) NOT trial.ti. or ("we searched".ab. AND (review.ti. OR review.pt.)) or "update review".ab. or (databases adj4 searched).ab. or (rat OR rats OR mouse OR mice OR swine OR porcine OR murine OR sheep OR lambs OR pigs OR piglets OR rabbit OR rabbits OR cat OR cats OR dog OR dogs OR cattle OR bovine OR monkey OR monkeys OR trout OR marmoset$1).ti. AND animal experiment/ or animal experiment/ NOT (human experiment/ OR human/))

*non-randomized studies on interventions*

#3 (exp cohort analysis/ or exp epidemiology/or exp clinical trial/or exp evaluation study/ or exp statistics/ or ((control and (group* or study)) or (time and factors) or program or survey* or ci or cohort or comparative stud* or evaluation studies or follow-up*).mp.) not ((animal/not human/) or editorial/or exp review/or meta analysis/or consensus/or exp practice guideline/ or case report.mp.)

condition (e.g. insomnia)

#4 exp sleep disorder/ or insomn*.ab,ti. OR sleep*.ab,ti. OR dyssomn*.ab,ti.

#5	#2 OR #3

#6	#1 AND #4 AND #5

## Cumulative Index to Nursing and Allied Health Literature (CINAHL) (Ebsco)

homeopathy

#1 ((MH "Homeopathy") OR (MH "Homeopathic Agents+") ) OR TI ( (homeopath* or homoeopath* or homoop* or omeop* or homopath* or potentis* or potentiz* or "materia medica" or nosode*) ) OR AB ( (homeopath* or homoeopath* or homoop* or omeop* or homopath* or potentis* or potentiz* or "materia medica" or nosode*) )

condition (e.g. insomnia)

#2 (MH "Sleep Disorders+") OR TI ( insomn* OR sleep* OR dyssomn* ) OR AB ( insomn* OR sleep* OR dyssomn* )

#3	#1 AND #2

## Cochrane Library (search manager)

homeopathy

#1	(homeopath* or homoeopath* or homoop* or omeop* or homopath* or potentis* or potentiz* or "materia medica" or nosode*):ti,ab,kw

condition (e.g. insomnia)

#2	(insomn* OR sleep* OR dyssomn*):ti,ab,kw

#3	#1 and #2

## Allied and Complementary Medicine Database (AMED)

homeopathy

#1 TI ( (homeopath* or homoeopath* or homoop* or omeop* or homopath* or potentis* or potentiz* or "materia medica" or nosode*) OR AB (homeopath* or homoeopath* or homoop* or omeop* or homopath* or potentis* or potentiz* or "materia medica" or nosode*)

condition (e.g. insomnia)

#2 TI ( insomn* OR sleep* OR dyssomn* ) OR AB ( insomn* OR sleep* OR dyssomn* )

#3           #1 AND #2

## LILACS (Advanced search)

(homeopath* OR homoeopath*) AND (insomn* or dyssomnia or Sleep Disorders) AND (randomly OR randomised OR randomized OR RCT OR controlled trial OR double blind OR placebo OR cohort OR observational OR longitudinal) [title, abstract, subject]

## PsycINFO (via Ebsco)

#1 TI ( (homeopath* or homoeopath* or homoop* or omeop* or homopath* or potentis* or potentiz* or "materia medica" or nosode*) ) OR AB ( (homeopath* or homoeopath* or homoop* or omeop* or homopath* or potentis* or potentiz* or "materia medica" or nosode*) )

#2 DE "Sleep Wake Disorders" OR DE "Insomnia OR TI ( insomn* OR sleep* OR dyssomn* ) OR AB ( insomn* OR sleep* OR dyssomn* )

#3	#1 and #2

## Web of Science

#1 ALL=(homeopath* or homoeopath* or homoop* or omeop* or homopath* or potentis* or potentiz* or "materia medica" or nosode*)

#2 ALL=(insomn* OR sleep* OR dyssomn* )

#3	#1 and #2

## HOMIS

“insomnia” in search field

## Trial register

[clinicaltrials.gov](http://www.clinicaltrials.gov)

Condition/disease: “insomnia”

Intervention/treatment: “homeopathy”

Study status: all studies

[trialsearch.who.int](http://www.trialsearch.who.int)

advanced search: “homeopath*” (title) AND “insomnia” (condition);

recruitment status: “ALL”

[clinicaltrialsregister.eu/ctr-search/search](http://www.clinicaltrialsregister.eu/ctr-search/search)

“homeopathy”

## Ayush Research Portal India

<https://ayushportal.nic.in/default.aspx>

medical system: “homeopathy”, category: “clinical research – evidence-grade A/B/C”, free text: “insomnia” in “article title”

## Google scholar

Homeopath* insomnia

## Preprint server

<https://osf.io/preprints>

Search field: “homeopathy”

<https://www.medrxiv.org/>

Search field: “homeopathy”

## OATD

<https://oatd.org/>

homeopath* AND (insomnia OR sleep)

## Proquest

[https://www.proquest.com/](https://www.proquest.com/advanced?accountid=98641)

abstract(homeopath* OR homoeopath* ) AND abstract(insomn* or dyssomnia or Sleep Disorders) AND (randomly OR randomised OR randomized OR RCT OR controlled trial OR double blind OR placebo OR cohort OR observational OR longitudinal)

# Search process documentation

Ovid MEDLINE(R) ALL <1946 to February 20, 2025>

1	Homeopathy/ or Materia Medica/ or Pharmacopoeias, Homeopathic as Topic/ or Formularies, Homeopathic as Topic/ or (homeopath* or homoeopath* or homoop* or omeop* or homopath* or potentis* or potentiz* or "materia medica" or nosode*).tw.	10780

2	(exp Randomized Controlled Trial/ or "controlled clinical trial".pt. or randomized.ab. or placebo.ab. or "Drug Therapy".fs. or randomly.ab. or trial.ab. or groups.ab.) not (exp animals/ not humans.sh.)	5440587

3	(exp cohort studies/ or exp epidemiologic studies/ or exp clinical trial/ or exp evaluation studies as topic/ or exp statistics as topic/ or ((control and (group* or study)) or (time and factors) or program or survey* or ci or cohort or comparative stud* or evaluation studies or follow-up*).mp.) not ((animals/ not humans/) or comment/ or editorial/ or exp review/ or meta analysis/ or consensus/ or exp guideline/ or hi.fs. or case report.mp.)	9582576

4	exp "Sleep Initiation and Maintenance Disorders"/ or insomn*.ab,ti. or sleep*.ab,ti. or dyssomn*.ab,ti.	276226

5	2 or 3	12091010

6	1 and 4 and 5	92

Embase Classic+Embase <1947 to 2025 February 20>

1	homeopathic agent/ or homeopathy/ or materia medica/ or (homeopath* or homoeopath* or homoop* or omeop* or homopath* or potentis* or potentiz* or "materia medica" or nosode*).tw.	19448

2	(exp randomized controlled trial/ or controlled clinical trial/ or random$.ti,ab. or randomization/ or intermethod comparison/ or placebo.ti,ab. or (compare or compared or comparison).ti,ab. or ((evaluated or evaluate or evaluating or assessed or assess) and (compare or compared or comparing or comparison)).ab. or (open adj label).ti,ab. or ((double or single or doubly or singly) adj (blind or blinded or blindly)).ti,ab. or double blind procedure/ or parallel group$1.ti,ab. or (crossover or cross over).ti,ab. or ((assign$ or match or matched or allocation) adj5 (alternate or group$1 or intervention$1 or patient$1 or subject$1 or participant$1)).ti,ab. or (assigned or allocated).ti,ab. or (controlled adj7 (study or design or trial)).ti,ab. or (volunteer or volunteers).ti,ab. or human experiment/ or trial.ti.) not ((((((((((((random$ adj sampl$ adj7 ("cross section$" or questionnaire$1 or survey$ or database$1)).ti,ab. not (comparative study/ or controlled study/ or randomi?ed controlled.ti,ab. or randomly assigned.ti,ab.)) or cross-sectional study/) not (exp randomized controlled trial/ or controlled clinical trial/ or controlled study/ or randomi?ed controlled.ti,ab. or control group$1.ti,ab.)) or ((case adj control$).mp. and random$.ti,ab.)) not randomi?ed controlled.ti,ab.) or (systematic review.ti,ab. not (trial or study).ti.) or (non random$ not random$).ti,ab. or "random field$".ti,ab. or (random cluster adj3 sample$).ti,ab. or (review.ab. and review.pt.)) not trial.ti.) or ("we searched".ab. and (review.ti. or review.pt.)) or "update review".ab. or (databases adj4 searched).ab. or (rat or rats or mouse or mice or swine or porcine or murine or sheep or lambs or pigs or piglets or rabbit or rabbits or cat or cats or dog or dogs or cattle or bovine or monkey or monkeys or trout or marmoset$1).ti.) and animal experiment/) or animal experiment/) not (human experiment/ or human/)) [mp=title, abstract, heading word, drug trade name, original title, device manufacturer, drug manufacturer, device trade name, keyword heading word, floating subheading word, candidate term word]	10258517

3	(exp cohort analysis/ or exp epidemiology/ or exp clinical trial/ or exp evaluation study/ or exp statistics/ or ((control and (group* or study)) or (time and factors) or program or survey* or ci or cohort or comparative stud* or evaluation studies or follow-up*).mp.) not ((animal/ not human/) or editorial/ or exp review/ or meta analysis/ or consensus/ or exp practice guideline/ or case report.mp.)	12711556

4	exp sleep disorder/ or insomn*.ab,ti. or sleep*.ab,ti. or dyssomn*.ab,ti.	579066

5	2 or 3	17850033

6	1 and 4 and 5	360

Cochrane Library (1948-20.2.2025)

ID	Search	Hits

#1	(homeopath* or homoeopath* or homoop* or omeop* or homopath* or potentis* or potentiz* or "materia medica" or nosode*):ti,ab,kw	2210

#2	(insomn* OR sleep* OR dyssomn*):ti,ab,kw	67025

#3	#1 and #2	99

CINAHL (1981 – 20.2.2025)

( ( (MH "Homeopathy") OR (MH "Homeopathic Agents+") ) OR TI ( (homeopath* or homoeopath* or homoop* or omeop* or homopath* or potentis* or potentiz* or "materia medica" or nosode*) ) OR AB ( (homeopath* or homoeopath* or homoop* or omeop* or homopath* or potentis* or potentiz* or "materia medica" or nosode*) ) ) AND ( (MH "Sleep Disorders+") OR TI ( insomn* OR sleep* OR dyssomn* ) OR AB ( insomn* OR sleep* OR dyssomn* ) )

LILACS (1988 – 20.2.2025)

(homeopath* OR homoeopath*) AND (insomn* or dyssomnia or Sleep Disorders) AND (randomly OR randomised OR randomized OR RCT OR controlled trial OR double blind OR placebo OR cohort OR observational OR longitudinal) [title, abstract, subject]

PsycINFO (1970-20.2.2025)

( ( (homeopath* or homoeopath* or homoop* or omeop* or homopath* or potentis* or potentiz* or "materia medica" or nosode*) ) OR AB ( (homeopath* or homoeopath* or homoop* or omeop* or homopath* or potentis* or potentiz* or "materia medica" or nosode*) ) ) AND ( DE "Sleep Wake Disorders" OR DE "Insomnia OR TI ( insomn* OR sleep* OR dyssomn* ) OR AB ( insomn* OR sleep* OR dyssomn* ) )

Web of Science (1900 – 20.2.2025)

(ALL=((homeopath* or homoeopath* or homoop* or omeop* or homopath* or potentis* or potentiz* or "materia medica" or nosode*))) AND ALL=((insomn* OR sleep* OR dyssomn* ))

AMED (1985 – 20.2.2025)

(TI ( (homeopath* or homoeopath* or homoop* or omeop* or homopath* or potentis* or potentiz* or "materia medica" or nosode*) OR AB (homeopath* or homoeopath* or homoop* or omeop* or homopath* or potentis* or potentiz* or "materia medica" or nosode*)) AND (TI ( insomn* OR sleep* OR dyssomn* ) OR AB ( insomn* OR sleep* OR dyssomn* ))
