# Supplementary material for: Homeopathy for the treatment of insomnia: a systematic review and meta-analysis
Source: Front Sleep. 2026 Sep 28;5:1922807. doi: 10.3389/frsle.2026.1922807 (PMC13654833; doi:10.3389/frsle.2026.1922807)
Supplement: Supplementary file 6 [file Data_Sheet_3.docx]

**Supplement 3**

## Study Eligibility Criteria

| **Domain** | **Include** | **Exclude** |
| --- | --- | --- |
| Population | Individuals with a primary diagnosis insomnia disorder of all ages (including adult and pediatric populations) and of both gender will be included. There will be no restrictions to mental, somatic or sleep comorbidities. | Individuals without a primary diagnosis of insomnia disorders |
| Intervention | Any type of homeopathic treatment involving the delivery of homeopathic preparations with all routes of administration | Interventions which do not match the definition of an HP |
| Comparator | any type of control (e.g. placebo, usual care, no treatment, active comparators) | Studies without a control, studies that compare 2 HPs, studies comparing combinations of HMPs and another intervention (e.g. herbal) with a third therapy |
| Outcome | Studies were required to report at least one insomnia-related clinical outcome measured at two or more time points (e.g., baseline and follow-up). Eligible outcomes included measures of insomnia severity, sleep quality, sleep parameters (e.g., sleep onset latency, total sleep time), and adverse events. | Studies reporting only economic outcomes or lacking outcome data relevant to insomnia were excluded. |
| Study characteristics | Randomized trials (RCTs) prospective, non‐randomized controlled studies of interventions (NRSIs). n-of-1 trials (in which a single participant receives both HMP and the control intervention in a randomly allocated sequence) if randomization and blinding are performed. | cross-sectional studies (without two or more points in time when outcomes are measured) retrospective cohort studies, case-control studies, before-after-studies (evaluation of a single group of participants at two time points), interrupted time series (as a special case of before-after-studies), case series, case reports, retracted publications. animal studies, study protocols |
