# Supplementary material for: Homeopathy for the treatment of insomnia: a systematic review and meta-analysis
Source: Front Sleep. 2026 Sep 28;5:1922807. doi: 10.3389/frsle.2026.1922807 (PMC13654833; doi:10.3389/frsle.2026.1922807)
Supplement: Supplementary file 7 [file Data_Sheet_4.docx]

**Supplement 4**

**Details of studies excluded after full-text screening, including reasons for exclusion, and Data Handling Decisions**

Table S.1: Ongoing studies

| **Study ID / Clinical trial** | **date registered** | **Status** | **Population** | **Planned N (total)** | **Intervention** | **Comparator** | **Co-intervention** |
| --- | --- | --- | --- | --- | --- | --- | --- |
| CTRI/2023/09/057225 | 04.09.2023 | Not Yet Recruiting | Insomnia | 100 | Individualized homoeopathy (centesimal potencies) | placebo | none |
| CTRI/2020/10/028710 | 28.10.2020 | Unknown | Insomnia | 60 | Individualized homoeopathy (centesimal potencies) | placebo | health advice (sleep hygiene, habits) |
| NCT02515006 | 30.07.2015 | Completed | Chronic insomnia in patients with cancer | 160 | homepathy | Unknown | Unknown |

https://trialsearch.who.int/Trial2.aspx?TrialID=CTRI/2023/09/057225

https://ctri.nic.in/Clinicaltrials/login.php

https://trialsearch.who.int/Trial2.aspx?TrialID=NCT02515006

Tab. S2: Authors contacted for additional information

| **study** | **date** | **Request** | **answer** |
| --- | --- | --- | --- |
| NCT02515006 | 21.02. | complete study | no answer |
| CTRI/2019/11/021931 | 21.02. | complete study | no answer |
| Hejazi, 2012 | 17.03.; 31.03. | email-adress of authors, additional information | no answer |
| Naude | 20.3. | SD values | provided raw data |
| Carlini, 1987 | 31.03. | SD values | no answer |
| Harrison, 2013 | 31.03. | SD values, p-values for quality of sleep, sleep onset latency | no access to original data |
| Waldschütz, 2008 | 01.04.; 07.04. | request for ITT-results and day 28 outcomes | no access to original data |
| Colombo, 2003 | 07.04. | request for additional baseline characteristics | no answer |
| Prosper, 2016 | 07.04. | general request for data | no answer |

Tab. S3: Studies excluded after full text screening with reasons

| **#** | **Reference** | **reason for exclusion** |
| --- | --- | --- |
| **1** | Survana B.S. (2012), Insomnia and homoeopathy, Natl J Homoeopath 2012;14():53-5 | wrong study design |
| **2** | van den Meerschaut, L., & Sünder, A. (2009). The Homeopathic Preparation Nervoheel N Can Offer an Alternative to Lorazepam Therapy for Mild Nervous Disorders. *Evidence-Based Complementary and Alternative Medicine*, *6*(4), 851249. | wrong patient population |
| **3** | La Pine, M. P., Malcomson, F. N., Torrance, J. M., & Marsh, N. V. (2006). Night shift: can a homeopathic remedy alleviate shift lag?. Dimensions of Critical Care Nursing, 25(3), 130-136. | wrong patient population |
| **4** | Hellhammer, J., & Schubert, M. (2013). Effects of a homeopathic combination remedy on the acute stress response, well-being, and sleep: a double-blind, randomized clinical trial. The Journal of Alternative and Complementary Medicine, 19(2), 161-169. | wrong patient population |
| **5** | Villet, S., Vacher, V., Colas, A., Danno, K., Masson, J. L., Marijnen, P., & Bordet, M. F. (2016). Open-label observational study of the homeopathic medicine Passiflora Compose for anxiety and sleep disorders. Homeopathy, 105(01), 84-91. | wrong patient population |
| **6** | Wolf, J. (1992), Schlafstörungen ohne hang-over behandeln: Ergebnisse einer klinischen Studie mit einem pflanzlich-homöopathischen Mittel, Natura Med. 1992;7(9):586-589 | wrong intervention |

Tab. S4: Data handling decisions

| **Study** | **Issue** | **Handling** |
| --- | --- | --- |
| Bell 2011 | Within-subject design | Mean difference extracted from regression model; included in sensitivity analyses only |
| Carlini 1987 | No variance information | Not included in meta-analysis |
| Harrison 2013 | Mixed reporting; no directly usable variance estimates | Sleep onset latency and sleep quality not included (insufficient data); for total sleep time, change approximated as regression slope × study duration and SD back-calculated from p-values of the linear regression model (not Friedman test); excluded in sensitivity analyses |
| Kolia-Adams 2010 | No SDs for change scores; test statistics reported | Standard errors derived from reported p-values and mean differences (Cochrane Handbook, Section 6.5.2.3); excluded in sensitivity analyses |
| Jong 2016 | Odds ratio reported | Transformed to SMD using Hasselblad & Hedges method |
| Michael 2019 | Change scores reported as baseline − post | Sign reversed for consistency |
| Naudé 2010 | Raw data incomplete/ambiguous | Not included in meta-analysis; reported narratively |
