# Supplementary material for: Homeopathy for the treatment of insomnia: a systematic review and meta-analysis
Source: Front Sleep. 2026 Sep 28;5:1922807. doi: 10.3389/frsle.2026.1922807 (PMC13654833; doi:10.3389/frsle.2026.1922807)
Supplement: Supplementary file 8 [file Data_Sheet_5.docx]

**Supplement 5**

Codebook

The codebook serves as a guide for reviewers when they are extracting information from the primary studies.

It was developed based on the Cochrane Handbook for Systematic Reviews of Intervention, chapter 5 [1], the practical guide of data extraction from Covidence [2], the CONSORT Harms 2022 statement [3], and under use of the Intervention Complexity Assessment Tool for Systematic Reviews (iCAT_SR) [4]. The glossary is a shortened version taken from CATHIS [5].

The codebook will be adapted, if necessary, according to the needs of the topic-specific systematic review.

# Additional guidance

- the Cochrane Handbook of Systematic Reviews, [chapter 5.5](https://training.cochrane.org/handbook/current/chapter-05#section-5-5),
- the template protocol of the series of systematic reviews,
- the framework paper,
- homeopathic pharmcopeias (excel file),
- the iCAT guidance.

# General extraction rules

- Whenever possible, please use the given category (e.g. enter “A” in the data extraction text field for study setting regarding the field “hospital”).
- Write “NA” for no information. Don’t leave blank fields.
- Please use the comments sections “notes” for additional information. Note anything that is relevant for extracting the corresponding fields and didn’t fit into the template.
- Use quotation marks if you copy and paste text directly from the study.
- Enter dates in the format “DD/MM/YYYY”
- Don’t contact study authors to obtain missing, not reported or unclear data without prior consultation with the review team to avoid redundant efforts.
- In case of questions that are not covered by the codebook, please contact:

[martin.loef@gkf-berlin.de](mailto:martin.loef@gkf-berlin.de)

# Identification

## **Study details**

### Sponsor

Extract the funding body (e.g. Homeopathy Research Institute, NA).

### Country

List the country or countries in which the study was conducted (e.g. Austria, NA)

### Setting

List the setting of the study (e.g. hospital, physicians (private practice), NA).

A: hospital

B: medical practice/office

C: outpatient center

D: nursing home

E: other (please specify under comments, e.g.: setting: home-based)

NA

### Sponsor category

Choose a category for the sponsor.

A: corporate

B: public

C: no funding

NA

### Publication type

Provide the type of publication.

A: journal article (peer-reviewed)

B: conference abstract

C: thesis

D: other: please specify (e.g: P4 (book chapter))

### Study start

Provide the year when the study started (e.g. 1984, NA).

### Study end

Provide the year when the study ended (e.g. 1987, NA).

### Conflict of interest

Did one or multiple of the authors declare a conflict of interest?

A: Yes

B: No

NA

### Notes

Please note anything that didn’t fit into the template.

# Methods

### Study design

- Enter the study design. If you choose “other”, please specify.
- Please specify whether the study has a parallel-group or a cross-over design.

The format is pre-specified by Covidence. For non-randomized studies of interventions, please differentiate between prospective controlled observational/cohort (choose in list) and prospective controlled experimental studies (type “exp” under “other”). If you are unsure about this decision, please read: <https://www.ncbi.nlm.nih.gov/books/NBK584468/>

### Centers

Enter whether the study is single or multi-center (single, multi center, NA).

A: single-center

B: multi-center

### Groups

Enter the number of treatment arms (e.g.: 3).

### Complexity

Please assess the following aspects of intervention complexity. Please also consider the guidance for using iCAT_SR for the rating [4].

#### Complexity 1

Define the number of active components in the intervention.

Details: Components can be defined as “the essential functions or principles, associated actors and processes and intervention activities that are judged necessary to produce desired outcomes” (doi: 10.1186/s12889-021-11244-3). Example: Rate A for a complex HMP, B for multiple HMPs or individualized therapy (single HMP plus patient-therapist interaction, and C for any type of homeopathy plus add-on. Don’t rate standard care as a component. See domain 1 of iCAT_SR.

A: 1 component

B: bundle^[1]^ of components

C: multiple components

#### Complexity 2

Define the degree of tailoring intended across individuals in applying the intervention.

Details: The degree of tailoring a homeopathic intervention is related to its individualization. Thus, complex, single non-isopathic, isopathic, restricted individualized (limited in the selection of homeopathic remedies), and unrestricted individualized homeopathy (not limited in the selection of remedies) are on the scale from “not individualized” to “fully individualized”. The degree of tailoring can be reflected in the flexibility of the intervention, but it can also be an inherent part of the study design (e.g., when a specific patient population receives a preparation that has been preselected for them through homeopathic individualization). See domain 4 of iCAT_SR.

A: clinical, complex/multi-constituents remedies

B: clinical, single-constituents (one remedy only)

C: clinical, isopathy

D: partly individualized (selection from predefined list or restricted change during time)

E: fully individualized

F: mixed

G: unclear

#### Complexity 3

Define the level of skill required by those delivering the intervention.

Details: The level of skill usually depends on the degree of individualization of a homeopathic intervention. If the intervention is not individualized, only basic skills may be required to deliver the HMP. Yet, high skills may be required for the entire therapy including co-therapies. See domain 5 of iCAT_SR.

A: basic skills

B: intermediate level skills

C: highly level skills

D: unclear/unable to assess

#### Complexity 4

Define the degree of interaction between intervention components such as the homeopathic remedy and the patient-therapist relation.

Details: The degree of interaction may depend on the degree of individualization of a homeopathic intervention. See domain 7 of iCAT_SR.

A. independent

B. moderate interaction

C. high level interaction

D. unclear/unable to assess

#### Complexity 5

Define the degree to which the effects of the interventions are changed by recipient or provider factors (e.g. A. patient expectations, culture and socioeconomic backgrounds, disease history, B. educational background, past experience). The dependence of individual-level factors usually dependence on the degree of individualization. For complex intervention a rating of B, for isopathy/clinical/routine homeopathy a rating of C and for classical homeopathy a rating of D may be most appropriate, but not imperative. See domain 9 of iCAT_SR.

A. largely independent of individual-level factors

B. moderately dependent of individual-level factors

C. highly dependent of individual-level factors

D. unclear/unable to assess

### ITT analysis

For RCT: Did the authors conduct an intention-to-treat analysis? (Enter NA for NRSI and “yes” if such an analysis is reported or all patients are included in the analysis).

A. yes

B. no

NA

### Reporting of harms in study methods

A: harms are prespecified and described, including how and when they were assessed AND the identification of non-prespecified harms is described

B: harms are prespecified and described, including how and when they were assessed

C. harms are prespecified and described, but without details of how and when they were assessed

D. harms are described in general

E. harms not described

### Reporting of harms in results

A: Both absolute and relative effect sizes for outcomes of harms are presented

B: Harms (excluding zero events) reported, but only with absolute or relative effect sizes, not both

C: Zero events are reported if no harms were observed

D: Harms not reported

E: other (please specify)

### Notes

Please note anything that didn’t fit into the template.

# Population

### Inclusion criteria

List all inclusion criteria (e.g.: “Children of both sexes, between 2 and 6 years of age. Earache of not more than 36 h duration.” [6]).

### Exclusion criteria

List all exclusion criteria.

### Group differences

Shortly state differences between the treatment arms regarding baseline characteristics (e.g.: no significant group differences between patient characteristics (see tab. 1), comorbidities (see page 335) and regarding symptoms or severity of illness (see page 335).)

### Sample size 1

Enter the number as integer of participants at baseline per treatment arm (e.g.: homeopathy: 233, control: 193).

### Sample size 2

Enter the number as integer of participants per treatment arm that were included in the analysis (e.g.: homeopathy: 233, control: 193).

### Unit of analysis

Provide the unit of analysis

A. individual participant

B. cluster of participants (e.g. clinics)

C. other (e.g. body part)

### Mean age

Enter the mean age with one decimal at baseline per treatment arm in years (e.g.: homeopathy: 35.7, control: 31.9).

### Gender

Enter the number of females as integer at baseline per treatment arm (e.g.: homeopathy: 123, control: 97).

### Treatment expectation

Were patients' expectations of treatment reported?

A. Yes

B. No

### Health beliefs

Were patients' health beliefs reported?

A. Yes

B. No

### Comorbidity

Do the patients suffer from further diseases and/or comorbidity?

A. Yes

B. No

NA

### Notes

Please note anything that didn’t fit into the template.

# Interventions

### Interventions

Homeopathy

Control

Interventions characteristics

### Goal

Please specify the intervention goal.

A. therapeutic

B. preventive

C. both

D. NA (Please specify).

### Potency level

Please specify the potency of the homeopathic treatment (e.g.: 30C, 50C, 200C, Q3-Q12).

### Potency type

Please specify the potency type.

A: Q/LM

B: D

C: C

D: MIX

NA

### Number of Consultation during study

Provide the number of consultations (or NA).

### Duration of consultation

Provide the duration of consultation (or NA).

### Add-on therapy

Was the homeopathic intervention used as add-on therapy?

A: yes

B: no

### Type of control intervention

Categorize the control treatment.

A: Placebo

B: Active

C: No treatment

D: other (please specify)

### Length of treatment

Enter the treatment duration as integer in days (convert weeks, months, years to 7, 30, 360 days).

### Length of follow-up

List the post-treatment follow-up in days as integer in days (convert weeks, months, years to 7, 30, 360 days).

### Notes

Please note anything that didn’t fit into the template.

# Outcomes

Describe outcomes

| Time points | Please state which time point you extract (e.g.: baseline). Choose the longest time point for the intervention duration of 4 weeks. In case of longer intervention durations and the availability of multiple time points, extract outcomes after 4 weeks and after maximum intervention duration if possible. |
| --- | --- |
| Scale | Define the scale you extract (e.g.: SF-36) |
| Range | Provide the the upper and lower limits of the scale (e.g.: 1-100). |
| Units | Please use the units (e.g. hours per night) provided. |
| Direction | Report whether higher or lower scores indicate improvement. |
| Data Value | Explain whether the data are change from baseline or endpoint scores. |
| Notes | Please note anything that didn’t fit into the template. In particular, note any calculations you conducted in any case that you did not extract original data reported in the study (e.g.: standard deviation for change from baseline calculated with the formula in Cochrane Handbook chapter 6.5.2.8 (2) based on SD(baseline), SD(end of study) and corr=0.5). |

### Specific guidance for outcome extraction

- If there are multiple time points and an effect estimate is reported that represents multiple time points (e.g. OR based on regression analysis accounting for repeated measurements), please extract this estimate.
- Otherwise, please select the longest follow-up of the outcome from each study if not stated differently (due to the relative clinical relevance of different time points).
- Covidence will provide entry fields for all arms. If you enter a mean difference between verum and control at the end of the study, please use the verum fields only and leave the control fields empty.
- Covidence offers data multiplicity for multiple time points, but not due to other reasons. If multiple measures of the same outcome are reported (e.g. IMOS answered by patients, IMOS answered by physicians), the time point fields may be used to differentiate between these groups.
- Covidence will provide entry fields for all arm. If you enter an OR, please provide the effect estimate for the verum group and set the reference group to “1”.

## Glossary

### **adverse drug reaction**

A response to a drug which is noxious and unintended and which occurs at doses normally used in man for prophylaxis, diagnosis, or therapy of disease or for modification of physiological function (GCP-V/ ICH-E2A [7]; WHO Technical Report 498 [1972])

### **adverse event**

Any untoward medical occurrence in a patient or clinical investigation subject administered a pharmaceutical product and which does not necessarily have to have a causal relationship with this treatment (GCP-V/ ICH-E2A [7]). The latter is contrary to adverse drug reactions, where causality with a medicinal product is suspected.

### **anthroposophically extended medicine**

A method to use homeopathic medicinal products for therapeutic purposes. It was developed by Rudolf Steiner, the founder of the philosophic and spiritual concept called ‘anthroposophy’.

### **antidotes to homeopathic prescriptions**

The potentially beneficial effect of a homeopathic medicinal product may be antidoted by several factors. There exists no complete list of antidotes; however, often cited are: coffee, camphor (to most of the vegetable medicines), etheric oils in ointments or hygiene products, medicated toiletries, herbal concoctions or medical drugs. It is therefore important to describe the handling of such antidotes during the clinical investigation, when reporting on homeopathic intervention studies. Additionally, obstacles to homeopathic cure or recovery are described in homeopathic textbooks. Their nature is rather universal (environmental or physical stressors to the human organism) and they are commonly not systematically assessed in intervention studies.

### **applicability of homeopathy to the condition**

As for the possible beneficial effect of homeopathy or homeopathic medicinal products (HMPs) for patients with the investigated condition, there may be several limitations to the applicability of homeopathy to the condition or HMPs respectively. These comprise among others: structural or anatomical changes or barriers which are causing the disease and call for a surgical or orthopaedic procedure, psycho-social or economic circumstances, language barriers leading to flaws in the homeopathic anamnesis or incomplete homoeopathic case documentation. A potentially beneficial effect may also be absent in case of incorrect preparation of the HMP (see production method of the homeopathic medicinal product) or incorrect homeopathic prescriptions (see indicated homeopathic medicine) as well as too frequent repetition of the HMP and/ or untimely/mistimed changes of the HMP during the course of treatment. Therefore, a preparation protocol of the HMP, a detailed dosage-protocol as well as the confidence in the homeopathic prescription are to be documented and the applicability to homeopathy is to be discussed, when reporting on homeopathic intervention studies.

### **assignment to intervention**

🡪 *see* *intention to treat*

### **Banerji-Protocol**

The Banerji Protocols are one homeopathic approach, in which specific, predefined combinations of homeopathic medicinal products are prescribed for a specific diagnosis of disease e.g. cancer, diabetes, asthma, eczema, COVID-19 etc.

### **baseline confounding**

Baseline confounding occurs when one or more prognostic variable(s), e.g. the factor(s) that predict the outcome of interest, also predicts the intervention received at baseline [8]. In other words, confounding arises when a factor is associated with both the exposure (or treatment) and the outcome.

### **clinical experience from homeopathic physicians**

Observations which homeopathic physicians or practitioners made over the years while approaching diseases with homeopathic care and especially with the individualised homeopathic strategy. In case a specific symptom of the patients disease leads to the individualised homeopathic prescription of a specific homeopathic medicinal product and the symptom disappeared after the administration of the medicine; this clinical experience adds to the homeopathic drug profile and may be upgraded in importance for further use of the applied medicine. This upgrade is usually registered in the homeopathic repertory.

### **clinical homeopathy**

The term defining the type of homeopathy, which uses routine homeopathic prescriptions for specific indications.

### **clinical relevance**

Besides statistical significance of a treatment effect, clinical relevance of the effect is important to clinicians. It describes the relevance of the investigated treatment in routine care (see also usual care) and can, thus, facilitate the interpretation of the research results into clinical practice. Important components of the clinical relevance of an intervention study are the sample size, the type of comparison intervention (see also control) and outcomes as well as the comparability of participants and setting and the intervention flexibility.

### **Coherence**

Besides methodological soundness (internal validity) and clinical relevance (external validity), coherence of the intervention with the principles, material, strategies and tools which define the intervention is an important quality-aspect of a clinical investigation: within intervention studies it is to be assured that the investigators tested what they wanted to test (model validity), i.e. that the clinical test was concordant with state-of-the-art procedures of the respective intervention. Coherence of clinical studies consists in an accurate, rational and referenced description of the intervention itself (e.g. which, when, how and by whom specific medicines or materials have been used), the current evidence base of the intervention, the specific research question as well as all strategies and tools used in the clinical study.

### **co-interventions**

All possible interventions, which are permitted or might occur alongside the investigated intervention and the comparison intervention during the participation in a clinical study. Permitted co-interventions may be any medical treatment needed for a chronic condition or any rescue medication indicated for the duration of the study. Additionally, co-interventions may occur, when the participants of the study are not fully compliant and use medical treatment other than the investigated and the comparison intervention by themselves. Permitted co-interventions are usually documented for the duration of a clinical study, whereas other co-interventions may not be documented and confound the results, especially in non-randomised studies.

### **complex homeopathic medicine**

A homeopathic medicinal product (HMP) prepared from more than one raw material combined into a single HMP (as opposed to a single homeopathic medicine). A synonym for this kind of HMP is multi-constituent HMP.

### **complex homeopathy**

The term defining the type of homeopathy using multi-constituent homeopathic medicinal products (HMPs) aka complex homeopathic medicines. Within complex homeopathy the HMPs are usually taken more than once daily and prescribed for predefined clinical indications.

### **confidence in the homeopathic prescription**

The individualised homeopathic strategy and some other homeopathic prescribing strategies consist in the identification of the plausible and indicated homeopathic medicine(s), which are to be applied for the individual patient in the specific clinical condition and situation. As each therapeutic situation demands an individual choice of the homeopathic medicinal product (HMP), the suitable potency and scale as well as a flexible frequency of application of the HMP, no generalizable homeopathic best-practice protocols exist. Makeshift, alongside referencing the individual homeopathic prescribing strategy, the confidence in the homeopathic prescription is an indicator for the prescription being the correct and indicated homeopathic medicine or not. Only, if the prescription was plausible, indicated and, thus, possibly correct, a potentially beneficial effect of the HMP under investigation can be expected. Therefore, it is important that the confidence in each prescription is documented when reporting on homeopathic intervention studies.

The confidence in the correct prescription is based on the concordance of the situation and the patients individual symptoms with the homeopathic drug profile, the information from homeopathic textbooks or based on the available references from clinical experience and scientific literature. Its documentation requires the experience of a homeopathic physician or practitioner.

### **Credibility**

Besides methodological soundness (internal validity), clinical relevance (external validity) and coherence of an intervention study, credibility has gained increasing attention. Credibility of a clinical investigation comprises the compliance of the clinical investigation with the principles of good clinical practice (GCP) and the reliability of the data. Reliability of the data can be assumed, if a research protocol is published before the start of the investigation, an ethical approval has been obtained, deviations from the protocol are reported and pseudonymised data is made available after completion of the trial.

### **formula composition**

The composition of a homeopathic formula. Within clinical studies, the rationale behind the formula composition should be stated (e.g. the indication of the compositions single components is referenced from the content of homeopathic textbooks or the rationale behind the composition of a complex homeopathic medicine is stated and referenced)

### **frequency table of the prescribed homeopathic medicines**

During a clinical study investigating individualised homeopathy, the intervention comprises several homeopathic medicinal products (HMPs), which are prescribed individually for each participant by a homeopathic physician or practitioner. A list, indicating how often and to what percentage a specific HMP has been used, allows to define the homeopathic intervention as detailed as possible.

### **Galenic**

Galenic is the process that turns an active ingredient into a ready-to-use medicine that can be dosed as required. Galenic formulation deals with the principles of preparing, compounding and dispensing medicines and forms part of pharmaceutics. As different galenic formulations may produce different therapeutic results it is important to state the form (e.g. suspension, tablets, oral drops) of a medicinal product, when reporting on intervention studies.

### **Hahnemannian method**

The original production method of a homeopathic medicinal product (HMP), which was developed by Samuel Hahnemann (1755–1843) and stated first in the Organon of Rational Therapeutics and further adapted and described in his later books. This method of preparation applies to the official homeopathic pharmacopoeia in Europe. It uses one glass vial per dilution step as compared to others (e.g. the Korsakovian-method), which use a total of only one glass vial during the preparation of the HMP. Due to expenditure of resources (glasses, ethanol and time), the Hahnemannian single glass method is not used for potencies over C1000.

### **Hering’s Law**

‘Hering’s Law of Cure’ is used by many homeopaths to help assess clinical outcomes. Although conceptually developed by Dr Constantine Hering, this instruction is an historical evolution of Samuel Hahnemann’s observations and thoughts regarding clinical changes occurring in patients after taking a homeopathic prescription. Hering’s Law stipulates that during a curative response to a prescription, symptoms improve in the following way: from the head down, from the inside out, from the most important to the least important organs, in the reverse order of symptoms that they first appeared. In clinical investigation they are preferably assessed according to the HELAT-flow chart [9].

### **homeopathic analysis strategies**

The method or strategy used to decide upon the specific homeopathic medicinal product(s), which is/are to be administered in a specific disease condition (indicated homeopathic medicine). As the respective strategies vary and may produce different results, it is important to describe the analysis strategy and reference its origin, when reporting on homeopathic intervention studies (e.g. description of the individualised homeopathic strategy or a reference for the homeopathic formula used in the clinical study). Examples for the individualised homeopathic strategy would be ‘Kentian’, ‘Boenninghausen’, or other homeopathic schools of thought, examples for references for homeopathic formula would be the respective textbooks or protocols (e.g. homotoxicology or the Banerji-Protocol).

### **homeopathic anamnesis**

One part of the homeopathic consultation. It consists in the recording of the evolutionary clinical history of the individual patient, including amongst other topics bio-psycho-social aspects, hereditary influences, medical antecedents and any treatments undertaken. As the intensity and duration of the interview is extended compared to a conventional doctor’s appointment, there exist claims that the homeopathic anamnesis by itself has a beneficial effect to many conditions and the administration of a homeopathic medicinal product (HMP) does not add to this effect. In clinical studies it is therefore important to distinguish between ‘homeopathic care’ (any homeopathic intervention, which includes a consultation with a homeopathic physician or practitioner) and the simple administration of HMPs. Further, it needs to be clearly stated, if both treatment groups received a homeopathic consultation or not.

### **homeopathic approach**

The specific method used to employ homeopathic medicinal products. See also type of homeopathy or homeopathic analysis strategies.

### **homeopathic care**

A form of homeopathic treatment comprising both, the homeopathic consultation and the application of one or more homeopathic medicinal products.

### **homeopathic consultation**

The homeopathic consultation consists of an in-depth interview of physical and psychological complaints of the patient (complete clinical case history aka homeopathic anamnesis) and the physical examination. The consultation concludes in the prescription of the indicated homeopathic medicine and is held by a homeopathic physician or practitioner. As the intensity and duration of the homeopathic consultation is extended compared to a conventional doctor’s appointment, there exist claims that the homeopathic consultation by itself has a beneficial effect to many conditions and the homeopathic medicinal product (HMP) does not add to this effect. In clinical studies it is therefore important to distinguish between ‘homeopathic care’ (any homeopathic intervention, which includes a consultation with a homeopathic physician or practitioner) and the simple administration of HMPs. Further, it needs to be clearly stated, if both treatment groups received a homeopathic consultation or not.

### **homeopathic dilution**

The term ‘homeopathic dilution’ is often used to describe the potency and scale of a homeopathic medicinal product. Though not specifically synonyms, the terms homeopathic dilution and homeopathic potency are sometimes used as such in literature.

### **homeopathic drug profile**

The summary of indicator symptoms for homeopathic medicines, which is used for the prescriptions of the homeopathic medicinal product for the individualised homeopathic prescription and routine homeopathic prescriptions by adhering to the ‘Law of Similars’.

### **homeopathic formula**

This term is used inconsistently either for single homeopathic medicinal products (HMPs) given in a prespecified pattern (formula) or for multi-constituent HMPs with a specific composition (formula). Multi-constituent HMPs with a specific composition are also referred to as patents.

### **homeopathic intervention studies**

Clinical studies investigating interventions with homeopathic medicinal products.

### **homeopathic medicinal product (HMP)**

Any medicinal product prepared from products, substances or compositions called homeopathic stocks in accordance with a homeopathic manufacturing procedure described by a homeopathic pharmacopoeia. The manufacturing process depends on the nature of raw or starting material (homeopathic stocks). It involves mostly in trituration of the raw material (grinding and diluting the substance in milk sugar) for low potencies, which is followed by a conversion into liquid potencies (dilutions) by diluting and succussing (vigorous shaking) for further potencies. For some stocks the herbal or animal raw material is extracted directly in ethanol or glycerol per maceration or percolation (mother tinctures) or dissolving the chemical substances in water/ ethanol. The tinctures are then, further diluted and succussed for the preparation of the homeopathic potencies.

The diluting ratio indicates the homeopathic scale: For so called ‘C-potencies’, the original substance is diluted stepwise 1:100, followed by at least 10 succussions; for so called ‘D’- or ‘X-potencies’, the original substance is diluted stepwise 1:10, followed by at least 10 succussions. For so called ‘Q’ or ‘LM-potencies’ (Fiftymillessimal potencies) the raw material is diluted 1:50’000 and succussed a 100 times during manufacturing. The number of production steps is indicated by the number of the potency (e.g. D12 or C12 are diluted 12 times with the corresponding ratio). In most cases the potentized liquid dilution is later applied to a sucrose or lactose vehicle (impregnated homeopathic granules).

Single homeopathic medicines are prepared from one single active substance and used for individualised homeopathic prescriptions and routine homeopathic prescriptions. So called multi-constituents or complex homeopathic medicines contain either more than one single homeopathic medicinal product or are directly prepared from more than one active substance. Multi-constituents are sometimes referred to as homeopathic formula or ‘patents’ (patented homeopathic formula).

As the number and the method of succussions differ between the national and regional homeopathic pharmacopoeia as well as between manufacturers, it is important to state the manufacturer of the investigational product (e.g. the HMP), the pharmacopoeia, or in absence thereof the preparation protocol used for the preparation of the HMP, when reporting on homeopathic intervention studies.

### **homeopathic nosode**

‘Nosode’ is the term used for a specific group of homeopathic medicinal products (HMPs). They are prepared from products of human or animal disease processes, from pathogens or their metabolic products, from the decomposition products of animal organs, or from cultured microorganisms [10]. The manufacturing processes are the standard methods for HMPs, but the homeopathic pharmacopoeias in current use include additional rules to produce safe, non-infectious starting material by different sterilisation methods. The subsequent serial dilution adds further safety and renders them as safe as other HMPs.

### **homeopathic pathogenetic trials**

Also known as ‘*homeopathic drug provings’;* a method to identify the indicator symptoms for homeopathic medicines. During the homeopathic pathogenetic trial, non-toxic levels of a specific substance or a homeopathic medicinal product are given to healthy volunteers and the symptoms exhibited by the subjects (responders only) are recorded. The profile of symptoms, recorded in a homeopathic pathogenetic trial, serve as basis to identify indicator symptoms for homeopathic medicines and form – together with clinical experience from homeopathic physicians the homeopathic drug profile summarized in the Materia Medica. The latter are used to treat patients with similar symptoms according to the ‘Law of Similars’.

### **homeopathic pharmacopoeia**

Compendium for homeopathic medicinal products (HMPs). It contains legally binding quality regulations for the raw and starting materials (homeopathic stocks), excipients, the final HMP and its preparation methods. In Europe it is comprised in the homeopathic part of the European Pharmacopoeia (Ph. Eur.) or, in the absence thereof, in the pharmacopoeias currently used officially in the Member States, i.e. German Homeopathic Pharmacopoeia (GHP) and French Pharmacopoeia (FP). In Asia, the binding compendium is the Homeopathic Pharmacopoeia of India (HPI), in the United States of America the Homeopathic Pharmacopoeia of the United States (HPUS) and in South America the Brazilian Homeopathic Pharmacopoeia (Brazil. HP) or, in the absence thereof, the pharmacopoeias currently used officially in the respective individual countries. As the monographs of the various homeopathic pharmacopoeias differ nationally or regionally (e.g. between Ph. Eur., GHP, FP, HPUS, HPI & Brazil. HP), it is important to state the pharmacopoeia used for the preparation of investigational product (e.g. the HMP), when reporting on homeopathic intervention studies.

### **homeopathic principles**

The theorems and declarations of the therapeutic approach of homeopathy and homeopathic care. They were founded by Samuel Hahnemann and are stated in the Organon of Rational Therapeutics or the Organon of the Healing Art respectively.

### **homeopathic schools**

This term describes the strategy used for the selection of the homeopathic medicinal product (HMP) of an individualised homeopathic prescription. Commonly known are for example the Kentian, Vithoulkas’, Shankaran’s or Sholten’s strategy or school. The different schools use different tactics or tools to identify the indicated homeopathic medicine. Therefore it is important to report the strategy used in a homeopathic intervention study.

### **homeopathic prescribing strategies**

The specific strategies used to choose the homeopathic medicinal product (HMP) to be administered to the patient. As opposed to conventional pharmacotherapy, where a specific pharmaceutical product is administered to every patient with the same clinical condition, homeopathy requires a selection of HMPs for each individual patient, situation and condition. Thus, various strategies to select and summarise the symptoms presented by the patient for the matching process with the indicator symptoms for homeopathic medicines or strategies to select complex homeopathic medicines exist. Examples are: the Boenninghausen , Kentian, or Shankaran’s method within individualised homeopathy, the theory and textbooks by Rudolf Steiner within anthroposophically extended medicine or the textbooks of Dr. Reckeweg within homotoxicology.

### **homeopathic textbooks**

Textbooks on various aspects of homeopathy, such as theory, principles of usage and application, pharmacology, the materia medica or repertories of indicator symptoms for homeopathic medicines. However, in scientific literature the materia medica is sometimes referred to as ‘homeopathic textbooks’. Thus, it is important that all textbooks or tools used for the respective homeopathic approach are referenced, when reporting on homeopathic intervention studies.

### **homeopathic tools**

The specific tools used to choose the homeopathic medicinal product to be administered to the patient. These may be repertories or homeopathic textbooks, or other compendia to support the respective homeopathic prescribing strategy.

### **homeopathy**

Is the term used to describe any therapeutic or preventive intervention with homeopathic medicinal products (HMPs). The original therapeutic method was developed in the late 18^th^ and the early 19^th^ century by Samuel Hahnemann. The basic principle is the Law of Similars, i.e. the selection and prescription of HMPs based on a matching process of the patients’ pathological symptoms with the indicator symptoms for homeopathic medicines or the homeopathic drug profile, as described in the Materia Medica, respectively. The intervention is supposed to regulate the organisms neuro-endocrinological, neuro-hormonal and neuro-immunological feed-back mechanisms and the resulting effect is expected to be observed in different functional levels of the organism, i.e. mental, emotional and physical symptoms may be affected at once [11-13].

Over the years, however, different homeopathic approaches and homeopathic prescribing strategies have been developed. They are commonly summarised and referred to as four type of homeopathy (see there). The resulting therapeutic effect of the respective homeopathic intervention changes presumably according to the strategy used. It is therefore important to describe the homeopathic prescribing strategies precisely, when reporting on homeopathic intervention studies.

### **homotoxicology**

A specific approach using several complex homeopathic medicines at once. It has been developed by Dr. Reckeweg. The theory behind this approach is that by the application of specially composed homeopathic medicinal products (aka antihomotoxins) the organism is drained of toxins and in consequence it’s immune system, endocrine or hormonal functioning is improved.

### **indicated homeopathic medicine**

The homeopathic medicinal product(s) selected by the homeopathic physician or practitioner, for each patient and situation individually according to the principles of homeopathy. For the individualised homeopathic strategy and routine homeopathic prescriptions one specific medicine is selected, whereas according to the theory of pluralistic homeopathic approaches or strategies applying homeopathic formula respectively, several homeopathic medicinal products deem indicated.

### **indicator symptoms for homeopathic medicines**

A profile of symptoms, exhibited by the patient, for which a specific homeopathic medicinal product (HMP) is indicated as a therapeutic intervention. The summary of indicator symptoms form the homeopathic drug profile, which is described in the homeopathic textbooks for each specific HMP. Sources of the indicator symptoms are homeopathic pathogenetic trials, the Materia Medica as well as clinical experience from homeopathic physicians. Both add to the repertory, where the indicator symptoms are indexed. Thus, the repertory usually facilitates the selection of the indicated homeopathic medicine.

### **individualised homeopathy**

One of the approaches or strategies to apply homeopathic medicinal products (HMPs). It is characterised by the use of only one of the HMPs at a time. The medicine to be given is identified after a homeopathic consultation or homeopathic anamnesis by matching all pathological symptoms exhibited by the patient with the indicator symptoms for homeopathic medicines or, respectively, the homeopathic drug profile, which is described in the Materia Medica. This matching process was defined by Samuel Hahnemann as the basic principle of homeopathy (Law of Similars). As the medicine is selected individually for each patient in accordance with the symptoms exhibited by the patient, this homeopathic approach is called ‘individualised homeopathy’ today. It is important to note that, for this strategy, the symptoms from different organs or functional systems are selected for the matching process (e.g. in a patient with diabetic foot ulcer and difficult breathing, the pathological symptoms of both organs are considered for the match).

### **initial aggravation**

One of the commonly known reactions to homeopathic medicinal products (HMPs). It is defined as a temporary worsening of existing symptoms following the administration of the indicated homeopathic medicine and is usually followed by an improvement. It is also called the ‘homeopathic aggravation’ [14]. There exists no agreement on the duration of the homeopathic aggravation and the handling of such an event demands professional experience (homeopathic training). In clinical studies, the homeopathic aggravation is usually recorded as an adverse event and, if prolonged or severe (i.e. in case of not indicated use of the investigated HMPs) also as an adverse drug reaction. The classification requires the assessment of an experienced homeopathic physician or practitioner.

### **Intention-to-treat**

The intention-to-treat principle defines that every patient included in the clinical study should enter the primary analysis. Accordingly, patients who drop out prematurely, are non-compliant to the study treatment, or even take the wrong study treatment, are included in the primary analysis within the respective treatment group they have been assigned to at randomisation (“as randomised”) or start of the study.

Consequently, in an analysis according to the ITT principle, the original randomization and the number of patients in the treatment groups remain unchanged, the analysis population is as complete as possible, and a potential bias due to exclusion of patients is avoided. Thus, the patient set used for the primary analysis according to the ITT principle is called ‘full analysis set’.

### **internal validity**

The extent to which the design and conduct of a study are likely to have prevented bias, whereas bias is a systematic error or deviation from the truth, in results or inferences [15].

### **intervention design**

The design according to which the interventions of clinical investigations are issued. Clinical studies may be designed with the investigated interventions administered alone or alongside other interventions (co-interventions). Or, the comparison interventions may be designed as an intervention following a standard protocol or they may be open and comply several permissible interventions. The latter is often the case, when the investigated intervention is compared to usual care treatment or a group of waiting-list subjects. All possible design features of the respective study should be described sufficiently in order to allow replication.

### **Isopathy**

A homeopathic approach consisting in the prescription of a homeopathic medicinal product, which is derived from the same substance which caused the disease (e.g. potencies of grass pollen for hay fever or potencies of arsen for chronic arsenic poisoning). With regard to terminology, isopathy is actually *no*t based on the Law of Similars (principle of likes cure likes) but on the notion that homeopathically prepared substances may cure diseases which have been caused by the very same substance. Therefore, isopathy is not *homeopathic* literally, but is mostly referred to as one of the homeopathic approaches [16, 17]. Moreover, there exist no clear distinction between isopathy and the administration of homeopathic nosodes, whereas the latter may sometimes be described with the term isopathy as well.

### **Korsakovian method**

One production method of the homeopathic medicinal product, which was invented by Simeon Korsakow (1787–1853) and named after him. For this method a total of only one glass vial is used during the preparation of the homeopathic medicinal product (HMP) and each dilution factor corresponds to a ratio 1:100. As compared to the Hahnemannian method, where one glass vial is used per dilution step, the Korsakovian method represents a considerable simplification and cost reduction. However, this production method of the HMP leads to slightly different degrees of dilution and different duration times of the HMP. As the Korsakovian method applies to the official homeopathic pharmacopoeia of the United States and the Brasilian homeopathic pharmacopoeia, whereas the Hahnemannian method applies to the homeopathic pharmacopoeia in Europe, it is important to state the pharmacopoeia used for the preparation of investigational product (e.g. the HMP), when reporting on homeopathic intervention studies.

### **Law of Similars**

The principle according to which homeopathic medicinal products are prescribed within the individualised homeopathic strategy. It was stated by Samuel Hahnemann, the founder of homeopathy in Latin: ‘similia similibus curentur’ (English: Let likes be treated with likes). It consists in the assumption that diseases can be treated with substances that induce similar symptoms in healthy persons (e.g. in homeopathic pathogenetic trials). This principle accounts for individualised homeopathy and routine homeopathic prescriptions.

🡪 *back to* Extended CONSORT checklist

🡪 *back to CATIS Template*

### **Materia Medica**

**Homeopathic textbooks** containing indicator symptoms for homeopathic medicines, which, taken together, form the homeopathic drug profile. Sources of these symptoms are homeopathic pathogenetic trials, clinical experience from homeopathic physicians as well as toxicology. The textbooks are used for individualised homeopathy and routine homeopathic prescriptions.

### **model validity**

The extent to which the design and conduct of a study are concordant with “state of the art” procedures [18]. In other words, the degree to which the model (population, intervention, comparator and outcome) tested in an intervention study is valid with regard to principles and gold standard practice of the investigated intervention.

### **obstacles to homeopathic cure**

In homeopathic textbooks several obstacles to recover from a disease are described. The comprise among others any potential stressors to the human organism, such as lack of sleep, extreme temperatures or overwork. Contrary to antidotes to homeopathic prescriptions these universal obstacles to recovery are difficult to assess within intervention studies and are rather subject to qualitative research or mixed-method research approaches.

### **Organon of Rational Therapeutics**

The book within which Samuel Hahnemann, the founder of homeopathy, constituted the code and principles for the correct preparation, selection and application of homeopathic medicinal products. The book is organised in aphorisms, which state the theoretical framework and the practical application of homeopathy. The first edition was published in 1810. From the second edition onwards, the name was changed into Organon of the Art of Healing. A total of 6 editions were published.

### **permitted changes to the homeopathic intervention**

Homeopathic treatment may comprise more than one homeopathic consultation (especially for chronic conditions). For many homeopathic prescribing strategies and especially for individualised homeopathy a change of the indicated homeopathic medicine is often needed when re-assessing the patient and condition during the follow-up consultations. In order to assess concordance with homeopathic practice (model validity), it should be reported within clinical studies whether the intervention was flexible concerning the choice of the homeopathic medicinal product, its dosage, potency and scale or not. That is to say, it should be reported, if changes to the homeopathic intervention were permitted within the study or not and, if permitted, when and how many times they were permitted.

### **permitted changes to the intervention**

The permission of changes in dosage or application frequency of the intervention as well as possible changes of the type of intervention describe the flexibility of the intervention: a strict protocol or regimen of the intervention, as often applied within clinical trials, does not always comply with the intervention as it is used in routine practice or usual care. Thus, using a strict protocol in clinical investigations may reduce clinical relevance, whereas permission of changes to the intervention, such as dosage- or frequency changes may better reflect usual care. In order to assess clinical relevance of the study results, it is important to know, whether changes to the intervention were permitted or not.

### **per protocol**

While an analysis according to the intention-to-treat (ITT) principle aims to preserve the original randomization and to avoid potential bias due to exclusion of patients, the aim of a per-protocol (PP) analysis is to identify a treatment effect which would occur under optimal conditions; i.e. to answer the question: what is the effect if patients are fully compliant (fully adhered to the intervention)? Therefore, some patients (from the full analysis set/ ITT-analysis) need to be excluded from the population used for the PP analysis (PP population).

### **pharmacopoeia**

The official compendium of medicines, published by the authority of a government or a medical or pharmaceutical society. It contains legally binding quality regulations for pharmaceuticals, excipients and some medical devices as well as methods for analysis and preparation of the latter.

### **pluralistic homeopathic approach**

A homeopathic prescribing strategy, using more than one single homeopathic medicine or more than one complex homeopathic medicine at a time.

### **potencies**

A potency characterises the homeopathic medicinal product (HMP) and defines, together with the denomination of the respective scale of the potency (see potency and scale), the HMP (see there). Depending on the dilution ratio of the original substance, the following terminology has been established: ‘C-potencies’ are prepared with the dilution ratio 1:100; ‘D’- or ‘X-potencies’ are prepared with the dilution ratio 1:10; and ‘Q’ or ‘LM-potencies’ are prepared with the dilution ratio 1:50’000. The manufacturing processes of the potencies are a stepwise trituration (grinding in milk sugar) or dilution in a liquid vehicle (mostly ethanol/ water mixtures) , followed by a certain number of succussions (process of vivid shaking). The number of potentisation steps is indicated by the number of the potency (e.g. D12 or C12 were potentised 12 times with the corresponding ratio).

### **potency and scale**

Potency and scale indicate the correct way of describing a homeopathic medicinal product (HMP). That is by the letters C, D or X, LM or Q indicating the *diluting ratio or scale* (e.g. so called ‘C-potencies’, where the original substance is diluted in a ration 1:100 or so called ‘D’- or ‘X-potencies’, where the original substance is diluted 1:10; see also HMP) and a number indicating the *potency*, i.e. the number of production steps, during which the dilution has been ‘succussed’ (vividly shaken). The potency C1000 is also described by the letter M (Latin for 1000), as well as C10.000 by XM, C50.000 by LM, C100.000 by CM and C1.000.000 by MM. Sometimes the production method of the homeopathic medicinal product is indicated by further letters, such as K or H (e.g. XMK for a HMP, which has the potency XM and was produced with the Korsakovian-method or 30CH for a homeopathic medicinal product, which has the potency C30 and was produced with the Hahnemannian method. Korsakovian potencies are sometimes only described by the letter K (e.g. 200K). Potencies over C1000 are mostly Korsakov potencies. The production method has to be mentioned, when reporting on homeopathic intervention studies.

### **production method of the homeopathic medicinal product**

The method used for the preparation of homeopathic medicinal products HMPs. The Hahnemannian method describes the original method of preparation and is the one logged in the official homeopathic pharmacopoeias in Europe. For this method of preparation one glass vial per diluting step is used, contrary to other methods, where one glass vial is used during the complete preparation (logged in the homeopathic pharmacopoeia of the United States and in the Brazilian homeopathic pharmacopoeia). Over the years, several methods how to dilute and succuss (vigorously shake) the HMP during the preparation have been developed. Examples are the Korsakovian-method, the Finke-method (Fluxion potencies) or others (see also potency and scale). As the effect and duration of action of the resulting HMP differs between the production methods, the manufacturer, the pharmacopoeia and/or if both are not applicable, the preparation protocol used for the HMP under investigation are to be described, when reporting on homeopathic intervention studies. This is particularly important, as production errors within the preparation of a HMP can lead to ineffective HMPs under investigation, which would cause major bias to the results of the respective clinical study.

### **prognosis or expected effect of the homeopathic intervention**

Depending on the specific clinical condition of a patient, as well as on the type of homeopathy and on the potency and scale used, the expected effect of the homeopathic intervention changes. For example are individualised homeopathic interventions supposed to regulate neuro-endocrinological, neuro-hormonal and neuro-immunological feed-back mechanisms of the organism and the resulting effect is expected to be observed in different functional levels of the organism, e.g. is expected to affect mental, emotional and physical functioning at once [11-13, 19] and potentially beneficial effects are observed in mental or emotional functions before changes of the physical symptoms appear (see also Hering’s Law). As these effects are not to be expected when complex homeopathy or a pluralistic homeopathic approach is applied, it is important that the type of outcome measurement complies with the expected effect of the respective homeopathic intervention and, if applicable, that the outcome measurements comprise tests for observing changes of all functional levels for the individualised homeopathic strategy.

Note: The expected effect of the homeopathic intervention also depends on the applicability of homeopathy to the condition to be investigated, the confidence in the homeopathic prescription and the absence of obstacles to homeopathic cure.

### **PROMS**

Patient-reported outcome measures (PROMs) are defined as standardised, validated questionnaires (which are also called instruments) completed by patients to measure their perception of their functional well-being and health status [20]. Patients rate their health by scoring the severity or difficulty in completing certain tasks or routine activities. PROM questionnaires are designed to be either generic or condition-specific.

### **proving symptom**

One of the commonly known reactions to homeopathic medicinal products (HMPs). It is defined as a *temporary* appearance of one of the indicator symptoms for the administered homeopathic medicine. Following the administration of the indicated homeopathic medicine the ‘proving symptom’ is usually followed by an improvement, whereas following the administration of a HMP which was not indicated (see also confidence in the homeopathic prescription), no improvement of the clinical condition follows. In clinical studies, a proving symptom is per definition recorded as an adverse event and, if unintended (i.e. in case of not indicated use of HMPs) also an adverse drug reaction.

### **reactions to homeopathic medicinal products**

Reactions to a homeopathic medicinal product (HMP) are most commonly defined by (a) a temporally limited aggravation of the symptoms for which the homeopathic medicinal products was prescribed (‘initial aggravation’), (b) a temporally limited appearance of symptoms described in the homeopathic medicinal textbooks for the administered homeopathic medicinal products (‘homeopathic proving symptom’), or (c) the appearance of new symptoms with synchronous disappearance of the symptoms for which the homeopathic medicinal products was prescribed (‘symptom-shift’). However, further reactions, such as feeling of tiredness [21] or changes in the immunological functioning of the organism (e.g. fevers) have been described [11].

In clinical studies all reactions to HMPs are recorded per definition as adverse events (AEs). The assessment, as to whether an AE is a reaction to a HMP and is classified as an adverse drug reaction requires homeopathic training [21].

### **RedHOT-criteria**

Standardised items to consider when “Reporting data on homeopathic treatments (RedHot)”. The criteria have been published as a supplement to CONSORT, developed with a Delphi-process from 12 homeopathic research experts [22] .

### **repertorisation**

The process of combining the individual symptoms of the patient and the respective homeopathic medicinal products (i.e. the indicator symptoms for homeopathic medicines) with help of the repertory, in order to identify and select the indicated homeopathic medicine.

### **repertory**

An analogue or digital index of indicator symptoms for homeopathic medicines, listing the possibly indicated homeopathic medicines for each symptom. Such index (software or print version) is used for the individualised homeopathic strategy to decide upon the indicated homeopathic medicine. As the content of different repertories vary, it is important to state the source of this important tool.

### **Risk of bias**

Risk of bias was defined as the risk of “a systematic error or deviation from the truth, in results or inferences” by the Cochrane Collaborations’ working group on quality of clinical studies [15]. It is interchangeable with internal validity, whereas low risk of bias defines high internal validity.

### **routine homeopathic prescriptions**

One of the approaches or strategies to apply homeopathic medicinal products (HMPs). It is characterised by the use of only one single homeopathic medicine at a time. The medicine to be given is identified after a short homeopathic evaluation by matching the apparent symptoms exhibited by the patient with the indicator symptoms for homeopathic medicines and/ or the homeopathic drug profile respectively. Thus, this method follows the Law of Similars. However, as opposed to the individualised homeopathic strategy, where all pathological symptoms of the patient are considered for the match, this strategy uses only symptoms of the acute or actual pathology of the patient. Additionally, only HMPs which are most commonly prescribed for the respective pathology are considered for the prescription.

### **routine practice**

The therapeutic approach of daily routine health care (see also usual care). As such, it comprises flexibility of therapeutic interventions with regard to patient needs, their situational condition as well as national laws or other regional circumstances (such as insurance plans or others).

### **symptom-shift**

One of the commonly known reactions to homeopathic medicinal products (HMPs). It is defined as the appearance of new symptoms with synchronous disappearance of the symptoms for which the HMP has been prescribed. If this symptom shift appears in accordance with Hering’s Law, i.e. from more dangerous or severe symptoms to less dangerous or severe symptoms, the reaction is considered to be favourable for the curative process after the application of the HMP. The duration of the new symptoms depends on several factors individual to the patient and the handling of such an event demands professional experience (homeopathic training). In clinical studies, the symptom-shift is per definition recorded as an adverse event (AE). The classification of the AE (e.g. the assessment of causality and severity) requires the evaluation of an experienced homeopathic physician or practitioner [21].

### **time-varying confounding**

In a clinical study, time-varying confounding can occur when individuals switch between the interventions being compared and when post-baseline prognostic factors affect the intervention received after baseline. If both is answered with *no* or *probably no* during the Cochrane risk of bias assessment [23], only baseline confounding needs to be assessed.

### **type of homeopathy**

The type of homeopathy describes one of the different homeopathic approaches or therapeutic strategies to apply homeopathic medicinal products (HMPs). In practice, there exist a great variety of strategies, which have been summarised under 4 categories for research purposes: individualised homeopathy, routine homeopathic prescriptions, multi-constituents or complex homeopathic medicine (also homeopathic formula or patents) and isopathy. Further important homeopathic prescribing strategies have been developed: amongst others, a so called pluralistic homeopathic approach, ‘homotoxicology’ (aka anti-homotoxicology), anthroposophically extended medicine, the so called biochemical approach by Dr. Schüssler and a method called ‘Spagyrik’.

### **usual care**

This term describes the type of care routinely received by patients in the geographic area in which the study is carried out. The care provided can vary by patient’s characteristics, the setting in which the patient is seen, individual providers and insurance plans [24].

References

1.	Li T, Higgins J, Deeks J. Chapter 5: Collecting data [last updated October 2019]. 2024. In: Cochrane Handbook for Systematic Reviews of Interventions version 65 [Internet]. Cochrane. Available from: <www.training.cochrane.org/handbook>.

2.	Covidence. A practical guide: Data Extraction for Intervention Systematic Reviews2024. Available from: <https://www.covidence.org/resource/data-extraction-for-intervention-systematic-reviews/>.

3.	Junqueira DR, Zorzela L, Golder S, Loke Y, Gagnier JJ, Julious SA, et al. CONSORT Harms 2022 statement, explanation, and elaboration: updated guideline for the reporting of harms in randomised trials. BMJ. 2023;381:e073725. doi: 10.1136/bmj-2022-073725.

4.	Lewin S, et al. Guidance for using the iCAT_SR: Intervention Complexity Assessment Tool for Systematic Reviews, Version 1.02016 14.01.2025. Available from: <https://methods.cochrane.org/sites/methods.cochrane.org/files/uploads/icat_sr_additional_file_4_2016_12_27.pdf>.

5.	Gaertner K, Hahn R, Razlog R, Musial F, Baumgartner S, Loef M, et al. Critical appraisal tool of homeopathic intervention studies – CATHIS. Complementary Medicine Research. 2024:under review.

6.	Sinha MN, Siddiqui VA, Nayak C, Singh V, Dixit R, Dewan D, et al. Randomized controlled pilot study to compare Homeopathy and Conventional therapy in Acute Otitis Media. Homeopathy. 2012;101(01):5-12. doi: 10.1016/j.homp.2011.08.003.

7.	European Medicines Agency - EMA. ICH Topic E 2 A - Clinical Safety Data Management: Definitions and Standards for Expedited Reporting.

8.	Kahlert J, Gribsholt SB, Gammelager H, Dekkers OM, Luta G. Control of confounding in the analysis phase - an overview for clinicians. Clin Epidemiol. 2017;9:195-204. Epub 2017/04/15. doi: 10.2147/CLEP.S129886. PubMed PMID: 28408854; PubMed Central PMCID: PMCPMC5384727.

9.	Harrison H. The development and use of Hering’s Law Assessment Tool (HELAT) in clinical trials. HRI Research Article [Internet]. 2012. Available from: <https://www.hri-research.org/wp-content/uploads/2014/09/HRI_ResearchArticle_18_Harrison_HELAT.pdf>.

10.	HMA. - Head of Medicine Agencies. Homeopathic Medicinal Products Working Group (HMPWG). List of Terms used in Homeopathy - related to homeopathic medicinal products [updated Novermber 2014; cited August 2020 August 2020]. Available from: <https://www.omeoimprese.it/wp-content/uploads/2015/01/2014_11_HMPWG_LIST_OF_TERMS_Adopted.pdf>.

11.	Vithoulkas G. Levels of Health - Second volume of the Science of Homeopathy. 3 ed. Homeopathy IAoC, editor. Alonissos: International Academy of Classical Homeopathy; 2019.

12.	Vithoulkas G. The Science of Homeopathy. 1 ed. New York, NY, United States of America: Grove Press; 1980.

13.	Vithoulkas G, Carlino S. The “continuum” of a unified theory of diseases. Med Sci Monit. 2010;16(2):SR7-15. PubMed Central PMCID: PMCPMID: 20110932.

14.	Stub T, Alraek T, Salamonsen A. The Red flag! risk assessment among medical homeopaths in Norway: a qualitative study. BMC Complement Altern Med. 2012;12:150. doi: 10.1186/1472-6882-12-150. PubMed PMID: 22967054; PubMed Central PMCID: PMCPMC3488491.

15.	Higgins JPT, S G. Cochrane Handbook for Systematic Reviews of Interventions Version 5.1.0 [updated March 2011]. The Cochrane Collaboration, 2011. Available from: <https://handbook-5-1.cochrane.org>.

16.	Dean ME. The Trials of Homeopathy. Essen: KVC-Verlag; 2004.

17.	Mathie RT, Hacke D, Clausen J, Nicolai T, Riley DS, Fisher P. Randomised controlled trials of homeopathy in humans: characterising the research journal literature for systematic review. Homeopathy. 2013;102(1):3-24. doi: 10.1016/j.homp.2012.10.002. PubMed PMID: 23290875.

18.	Bornhoft G, Maxion-Bergemann S, Wolf U, Kienle GS, Michalsen A, Vollmar HC, et al. Checklist for the qualitative evaluation of clinical studies with particular focus on external validity and model validity. BMC Med Res Methodol. 2006;6:56. doi: 10.1186/1471-2288-6-56. PubMed PMID: 17156475; PubMed Central PMCID: PMCPMC1764896.

19.	Oberbaum M, Vithoulkas G, Van Haselen R. Clinical trials of classical homeopathy: reflections on appropriate research designs. J Altern Complement Med. 2003;9(1):105-11. doi: 10.1089/107628003322017323. PubMed PMID: 12676039.

20.	National Health Service (NHS), Department of Health. Guidance on the routine collection of Patient Reported Outcome Measures ( PROMs ). [Internet].2009.

21.	Stub T, Salamonsen A, Alraek T. Is it possible to distinguish homeopathic aggravation from adverse effects? A qualitative study. Forsch Komplementmed. 2012;19(1):13-9. Epub 2012/03/09. doi: 10.1159/000335827. PubMed PMID: 22398921.

22.	Dean ME, Coulter MK, Fisher P, Jobst K, Walach H. Reporting data on homeopathic treatments (RedHot): a supplement to CONSORT. Homeopathy. 2007;96(1):42-5. doi: 10.1016/j.homp.2006.11.006. PubMed PMID: 17227747.

23.	Sterne JA, Hernan MA, Reeves BC, Savovic J, Berkman ND, Viswanathan M, et al. ROBINS-I: a tool for assessing risk of bias in non-randomised studies of interventions. BMJ. 2016;355:i4919. Epub 2016/10/14. doi: 10.1136/bmj.i4919. PubMed PMID: 27733354; PubMed Central PMCID: PMCPMC5062054 <http://www.icmje.org/coi_disclosure.pdf> and declare: grants from Cochrane, MRC, and NIHR during the conduct of the study. Dr Carpenter reports personal fees from Pfizer, grants and non-financial support from GSK and grants from Novartis, outside the submitted work. Dr Reeves is a co-convenor of the Cochrane Non-Randomised Studies Methods Group. The authors report no other relationships or activities that could appear to have influenced the submitted work.

24.	Wieland LS, Berman BM, Altman DG, Barth J, Bouter LM, D'Adamo CR, et al. Rating of Included Trials on the Efficacy-Effectiveness Spectrum: development of a new tool for systematic reviews. J Clin Epidemiol. 2017;84:95-104. Epub 2017/02/12. doi: 10.1016/j.jclinepi.2017.01.010. PubMed PMID: 28188898; PubMed Central PMCID: PMCPMC5441969.

1. “A bundle is a set of intervention components that are intended to be used together to improve patient outcomes.” (see Lewin et al. (2016))
