# Supplementary material for: Homeopathy for the treatment of insomnia: a systematic review and meta-analysis
Source: Front Sleep. 2026 Sep 28;5:1922807. doi: 10.3389/frsle.2026.1922807 (PMC13654833; doi:10.3389/frsle.2026.1922807)
Supplement: Supplementary file 9 [file Data_Sheet_8.docx]

**Supplement 8:**

Risk of Bias and Meta-analysis

## 1. Quality of sleep

Fig. 1a: Homeopathy vs. placebo for insomnia in adults, outcome: quality of sleep (Pittsburgh sleep quality index), MD<0 indicates relative benefit of HP vs. placebo

† individualized homeopathy

Fig. 1b: RoB-2 for randomized controlled trials regarding sleep quality in adults treated with homeopathy

## 2. Total sleep duration

Fig. 2a: Homeopathy vs. placebo for insomnia in adults, outcome: total sleep duration in hours, MD>0 indicates relative benefit of HP vs. placebo

† individualized homeopathy

Fig. 2b: RoB-2 for randomized controlled trials regarding total sleep time in adults treated with homeopathy

Fig. 2c: RoB-2 for crossover trials* regarding total sleep time and sleep quality. Carlini is not included in the meta-analysis due to missing data; *Bell (2011) is a NRSI including a within-subject crossover design that is only used for a sensitivity analysis.

Fig. 2d: ROBINS-I V2 for non-randomized studies on total sleep duration in adults

## 3. Insomnia severity

Fig. 3a: Homeopathy vs. placebo for insomnia in adults, outcome: insomnia severity, SMD>0 indicates relative benefit of homeopathy vs. placebo

† individualized homeopathy

Fig. 3b: RoB-2 for randomized controlled trials regarding insomnia severity in adults treated with homeopathy

## 4. Sleep onset latency

Fig. 4a: Homeopathy vs. placebo for insomnia in adults, outcome: sleep onset latency in minutes, MD<0 indicates relative benefit of HP vs. placebo

† individualized homeopathy

Fig. 4b: RoB-2 for randomized controlled trials regarding sleep onset latency in adults treated with homeopathy

## 5. Sleep efficiency

Fig. 5a: Homeopathy vs. placebo for insomnia in adults, outcome: sleep efficiency, MD>0 indicates relative benefit of HP vs. placebo

† individualized homeopathy

Fig. 5b: RoB-2 for randomized controlled trials regarding sleep efficiency in adults treated with homeopathy

## 6. Insomnia severity (children)

Fig. 6a: Homeopathy vs. placebo for insomnia in children, outcome: insomnia severity, SMD>0 indicates relative benefit of HP vs. placebo

Fig. 6b: RoB-2 for randomized controlled trials regarding insomnia severity in children with glycine as control interventions

## 7. Adverse events

Fig. 7a: RoB-2 for randomized controlled trials regarding adverse events in adults with placebo as control intervention

Fig. 7b: ROBINS-I V2 for non-randomized studies on adverse events in adults

Fig. 7c: RoB-2 for randomized controlled trials regarding adverse events in children with glycine as control intervention

## 8. Exploratory and sensitivity analyses

Fig. 8: Exploratory analysis: Homeopathy vs. placebo for insomnia in adults, outcome: overall symptom reduction,multilevel meta-analysis with intra-class correlation coefficient rho=0.5, SMD>0 indicates relative benefit of HP vs. placebo

† individualized homeopathy

Fig. 9: Sensitivity analysis, Homeopathy vs. placebo for insomnia in adults, outcome: quality of sleep (Pittsburgh sleep quality index), MD<0 indicates relative benefit of HP vs. placebo, with Bell (2011)

† individualized homeopathy

Fig. 10: Sensitivity analysis, Homeopathy vs. placebo for insomnia in adults, outcome: total sleep duration in hours, MD>0 indicates relative benefit of HP vs. placebo; without studies with data-imputation according to supp. 4

† individualized homeopathy

Fig. 11: Sensitivity analysis, Homeopathy vs. placebo for insomnia in adults, outcome: total sleep duration in hours, MD>0 indicates relative benefit of HP vs. placebo; with Bell (2011)

† individualized homeopathy

Fig. 12: Sensitivity analysis, Homeopathy vs. placebo for insomnia in adults, outcome: total sleep duration in hours, MD>0 indicates relative benefit of HP vs. placebo; low/moderate risk of bias

† individualized homeopathy

Fig. 13a: Sensitivity analysis of exploratory analysis: Homeopathy vs. placebo for insomnia in adults, outcome: overall symptom reduction, multilevel meta-analysis with intra-class correlation coefficient rho=0, SMD>0 indicates relative benefit of HP vs. placebo

† individualized homeopathy

Fig. 13b: Sensitivity analysis of exploratory analysis: Homeopathy vs. placebo for insomnia in adults, outcome: overall symptom reduction, multilevel meta-analysis with intra-class correlation coefficient rho=0.9, SMD>0 indicates relative benefit of HP vs. placebo

† individualized homeopathy

Fig. 13c: Sensitivity analysis of exploratory analysis: Homeopathy vs. placebo for insomnia in adults, outcome: overall symptom reduction, multilevel meta-analysis with intra-class correlation coefficient rho=0.5, SMD>0 indicates relative benefit of HP vs. placebo; only studies with low/moderate risk of bias

† individualized homeopathy

Fig. 14: Funnel plot to meta-analysis in figure 2: Homeopathy vs. placebo for insomnia in adults, outcome: total sleep duration in hours, MD>0 indicates relative benefit of HP vs. placebo

Fig. 15: Funnel plot to meta-analysis in figure 7: Homeopathy vs. placebo for insomnia in adults, outcome: total sleep duration in hours, MD>0 indicates relative benefit of HP vs. placebo

| **Study** | **AE Monitoring Method** | **Homeopathic Aggravations** | **Any Adverse Events** | **Serious Adverse Events** | **tolerance** |
| --- | --- | --- | --- | --- | --- |
| Jong et al. (2016) | AE monitoring explicitly mentioned; assessed at follow-up visits and final evaluation by both investigator and parents using a 4-point scale. | Not reported. | 11 AEs total: 6 in verum (ZinCyp-3-02), 5 in control. All mild/moderate. Verum: 4 URTIs (not related), 1 nervousness (possibly related), 1 excitability (unlikely related, led to withdrawal). Control: 2 URTIs, 1 otalgia, 1 food allergy (all not related), 1 excitement (probably related). | None. | rated as “very good” (ZinCyp-3-02: 62-63 out of 89 children, control: 58-59 out of 90) or “good” (ZinCyp-3-02: 25-26 out of 89, control: 25-26 out of 90) by most children |
| Michael et al. (2019) | Rescue remedy protocol planned for AE/SAE; adverse events noted during monthly follow-ups; outcomes monitored by blinded investigators. | Not reported. | One case of rectal bleeding (not serious, unclear in which group), managed homeopathically. | None. | Not reported. |
| Waldschütz & Klein (2008) | Adverse events were explicitly recorded; tolerability rated by physicians via patient dialogue (scale: excellent to poor). | Not reported. | One case of mild caffeine intolerance in Neurexan group. | None. | rated as “excellent” by 90% of subjects treated with Neurexan and 89% of subjects treated with valerian preparations. |

Tab. S8.1: Reporting and monitoring of adverse events in included studies
