# Supplementary material for: Homeopathy for the treatment of insomnia: a systematic review and meta-analysis
Source: Front Sleep. 2026 Sep 28;5:1922807. doi: 10.3389/frsle.2026.1922807 (PMC13654833; doi:10.3389/frsle.2026.1922807)
Supplement: Supplementary file 10 [file Data_Sheet_9.docx]

**Supplement 9**

**MVHT Consensus**

| Study | Domain I | Domain II | Domain III | Domain IV | Domain V | Domain VI | Overall MVHT |
| --- | --- | --- | --- | --- | --- | --- | --- |
| Carlini 1987 | Unclear | Unclear | Yes | Unclear | Yes | Yes | Uncertain |
| Harrison 2013 | Unclear | Unclear | Yes | Yes | Yes | Yes | Uncertain |
| Hejaji 2012 | Yes | Yes | Unclear | Yes | Unclear | Yes | Uncertain |
| Jong 2016 | Unclear | Unclear | Unclear | Yes | Yes | Yes | Uncertain |
| Kolia-Adam 2010 | Yes | Yes | Yes | Yes | Yes | Yes | Acceptable |
| Michael 2019 | Yes | Yes | Yes | Yes | Yes | Yes | Acceptable |
| Naudé 2010 | Yes | Yes | Unclear | Yes | Yes | Yes | Acceptable |
| Prajakta 2024 | Yes | Yes | Unclear | Yes | Yes | Yes | Acceptable |
| Bell 2011 | Yes | Yes | Yes | Yes | Yes | Yes | Acceptable |
| Colombo 2003 | Unclear | No | Yes | Yes | Yes | Yes | Inadequate |
| Prosper 2016 | Unclear | Unclear | Unclear | Unclear | Unclear | Yes | Uncertain |
| Waldschuetz 2008 | Unclear | Unclear | No | Yes | Yes | Yes | Inadequate |

**RITES Consensus**

| Study | Participants’ Characteristics ( | Trial Setting | Flexibility of Interventions | Clinical Relevance of Interventions | Average Score |
| --- | --- | --- | --- | --- | --- |
| Carlini 1987 | 4 | 5 | 5 | 2 | 4 |
| Harisson 2013 | 1 | 3 | 2 | 1 | 1.75 |
| Hejazi 2012 | 2 | 1 | 1 | 3 | 1.75 |
| Jong 2016 | 2 | 1 | 1 | 2 | 1.5 |
| Kolia-Adam 2010 | 1 | 1 | 2 | 1 | 1.25 |
| Michael 2019 | 2 | 2 | 5 | 1 | 2.5 |
| Naudé 2010 | 4 | 1 | 4 | 1 | 2.5 |
| Prajakta 2024 | 1 | 4 | 5 | 1 | 2.75 |
| Bell 2011 | 1 | 1 | 1 | 1 | 1 |
| Colombo 2003 | 2 | 5 | 1 | 5 | 3.25 |
| Prosper 2016 | 5 | 5 | 4 | 4 | 4.5 |
| Waldschuetz 2008 | 4 | 5 | 2 | 4 | 3.75 |
